# Conditional Drug Approval as a Path to Market for Oncology Drugs in Canada: Challenges and Recommendations for Assessing Eligibility and Regulatory Responsiveness

**DOI:** 10.3389/fmed.2021.818647

**Published:** 2022-02-03

**Authors:** Melanie McPhail, Emma Weiss, Tania Bubela

**Affiliations:** Faculty of Health Sciences, Simon Fraser University, Burnaby, BC, Canada

**Keywords:** conditional regulatory approval, drug regulation, oncology, unmet medical need, lifecycle regulation

## Abstract

International drug regulators use conditional drug approval mechanisms to facilitate faster patient access to drugs based on a lower evidentiary standard typically required of drug approvals. Faster and earlier access is justified by limiting eligibility to drugs intended for serious and life-threatening diseases and by requiring post-market evidence collection to confirm clinical benefit. One such mechanism in Canada, the Notice of Compliance with Conditions (NOC/c) policy, was introduced in 1998. Today, most of the drugs approved under the NOC/c policy are for oncology indications. We analyze oncology drugs approvals under the NOC/c policy to inform discussions of two tradeoffs applied to conditional drug approvals, eligibility criteria and post-market evidence. Our analysis informs recommendations for Canada's proposed regulatory reforms approach to conditional approvals pathways. Our analysis demonstrates that under the current policy, eligibility criteria are insufficiently defined, resulting in their inconsistent application by Health Canada. Regulatory responsiveness to post-market evidence from post-market clinical trial and foreign jurisdiction regulatory decisions is slow and insufficient. In the absence of sufficient regulatory responsiveness, physicians and patients must make clinical decisions without the benefit of the best available evidence. Together, our analysis of the two core tradeoffs in Canada's conditional drug approval provides insight to inform the further development of Canada's proposed agile regulatory approach to drugs and devices that will expand the use of terms and conditions.

## Introduction

Drug regulators have introduced conditional drug approval pathways to accelerate patient access to promising therapies since the 1990s. Though drugs approved under accelerated or conditional pathways must still demonstrate a positive benefit-risk balance, approvals are based on lower evidentiary standards than standard regulatory approval processes that evaluate safety and efficacy (1–3). Due to the heightened risk to patients associated with approving drugs on earlier evidence, conditional pathways are justified by limiting eligibility, for example, to drugs intended for serious and life-threatening diseases or where there is unmet need (4). They further require post-market evidence collection to confirm clinical benefit. Accelerated and conditional approval pathways provide an important channel for drugs to gain market access that may be precluded under traditional regulatory approaches. However, conditional drug approval pathways have drawn criticism for lack of timely and appropriate enforcement, lack of transparency in their application and processes, erosion of evidentiary thresholds, loss of institutional trust, and increased patient risk without proportionate justification (5–12).

Conditional drug approval mechanisms introduce greater potential for risk by permitting increased flexibility in pre-market evidence requirements and greater tolerance for uncertainty. Studies in Canada and the US have found that drugs approved under accelerated and conditional regulatory pathways are more likely to receive post-market safety warnings (8, 13). This increased risk is justified by post-market evidence collection commitments and by limiting eligibility for conditional approval to drugs and indications where patients may be willing to tolerate risk. Rather than making patients wait years to access a promising therapy, conditional approval pathways permit patients to benefit months or years earlier than they might be able to if the drug were required to meet traditional regulatory evidence standards. The potential for patients to benefit from a promising therapy justifies the added risks associated with the lower evidence threshold at the time of approval. However, defining eligibility criteria to ensure that only appropriate drugs are approved conditionally has proved challenging. A review of drugs approved under expedited pathways in Canada found that Health Canada's ability to identify promising drugs that offer major therapeutic benefit is limited, suggesting that the pathways are not fulfilling their expectations (14).

Here, we evaluate approvals under Canada's conditional drug approval pathway—the Notice of Compliance with Conditions (NOC/c) policy (15) to inform discussions on eligibility criteria and post-market evidence, administered by Canada's drug regulator, Health Canada. Our analysis is timely, because Health Canada recently announced consultations on proposed reforms toward agile regulation for drugs (16). We limit the scope of our analysis to oncology drugs, which comprise the majority of recent approvals under the policy (70% of approvals).

We first outline the conditional regulatory approval pathway in Canada under the NOC/c policy. We then describe our analytical approach and data acquisition, followed by our findings on two regulatory challenges: (1) the application of unmet medical need as an eligibility criterion, defined as either no existing therapy or an improvement over an existing therapy; and (2) the regulatory responsiveness of Health Canada to post-market evidence, including evidence of regulatory action in other jurisdictions, the status of post-market clinical trials, and label changes following the submission of results from confirmatory trials. Based on our analyses, we then consider how the current NOC/c pathway can inform the development of the proposed agile regulatory approach. Finally, we discuss the limitations of our analysis and conclude that appropriate eligibility criteria and enforcement of post-market commitments will enable the proposed expanded use of post-market terms and conditions.

## Conditional Regulatory Approval In Canada

Health Canada introduced its NOC/c policy in May 1998, following the lead of the United States (US) Food and Drug Administration's (FDA) Accelerated Approval (AA) program. The NOC/c policy is not enshrined in either the *Food and Drugs Act* (17) or the *Food and Drugs Regulations* (*FDR*) (18). Instead, the NOC/c pathway was implemented through the *NOC/c Policy Guidance Document* [Guidance Document], a non-legally binding instrument.

Like other accelerated approval pathways, the goals of the NOC/c policy are to facilitate earlier access to drugs and to permit enhanced post-market surveillance to monitor the safety and efficacy of promising new therapies (15). To meet these goals, eligible drugs need to demonstrate promising evidence of efficacy, be indicated for a serious, life-threatening or severely debilitating disease or condition, and address an unmet medical need, represented by either absence of an available therapy or significant improvement over existing therapies (15). In comparison, drugs approved under the standard approval pathway must demonstrate “substantial evidence of clinical effectiveness (18).” which typically requires two well-controlled trials (19).

According to the Guidance Document, Health Canada has flexibility and discretion to assess whether the eligibility criteria are met. It can construe the promising nature of the drug from “[t]rials with surrogate markers that require validation; Phase II trials that would require confirmation with Phase III trials…; [or] Phase III trials where a single small to moderately sized trial would require confirmation (15). It also has discretion over whether a drug meets either the serious or life-threatening disease or severely debilitating disease threshold, although some diseases are explicitly listed as serious conditions, including HIV/AIDS, amyotrophic lateral sclerosis, and cancer. Severely debilitating diseases may include chronic conditions such as inflammatory bowel disease, asthma, depression, and rheumatoid arthritis. In contrast, the NOC/c Policy does not specify how Health Canada determines whether an existing therapy is available. Instead, it provides guidance on acceptable metrics for whether a drug provides an improvement in benefit/risk profile over an existing therapy (15).

Under the NOC/c policy, sponsors must undertake to carry out additional clinical trials to verify the clinical benefit, defined as “outcomes that have an overall positive impact on the treatment of a disease (15).” Other undertakings include enhanced monitoring and reporting to Health Canada; provision of educational materials to healthcare practitioners and patients; and restrictions on advertising and labeling. Once a sponsor provides Health Canada with satisfactory evidence of the drug's clinical effectiveness, and Health Canada is satisfied that all conditions have been met, Health Canada will remove the conditions (15). However, until confirmation of clinical benefit, public and private drug plans may or may not cover the costs of drugs authorized under the NOC/c Policy.

Health Canada issues a Qualifying Notice to drugs that have successfully navigated the NOC/c process, which outlines the additional clinical evidence to be provided in confirmatory studies, post-market surveillance responsibilities, and requirements related to advertising, labeling, or distribution. The sponsor must respond with the final Letter of Undertaking (LoU), which contains details on how the Sponsor will meet the conditions in four domains: well-designed confirmatory trials to demonstrate clinical benefit; heightened post-market surveillance and reporting on safety and effectiveness, including whether actions have been taken in a foreign jurisdiction; details related to product monographs, labeling and consumer information; and compliance with restrictions on advertising or distribution. Once Health Canada finalizes conditions specified in the LoU, it will issue a Notice of Market Authorization with Conditions, which highlights the conditional nature of the authorization and is communicated through Health Canada's Health Product InfoWatch, a monthly regulatory publication intended for health professionals (20). Submission of results from confirmatory trials within the agreed-to timeframe results in the transferal of the NOC/c approval to a standard NOC. However, if all undertakings are not satisfied, or the sponsor foresees an inability to adhere to the agreed upon timelines, a new LoU must be submitted along with a letter that provides the rationale for the changes (15).

Health Canada can use its enforcement capabilities in three circumstances set out in the *FDR* when sponsors fail to comply with any of the undertakings contained in the LoU. First, if the evidence submitted as required by a LoU is not sufficient, Health Canada may notify the sponsor and prohibit it from selling the drug until sufficient evidence is submitted (15, 18). Second, Health Canada may suspend the NOC/c if the confirmatory trials fail to demonstrate clinical benefit or if the confirmatory trials raise safety concerns (18). Third, Health Canada can take action if the sponsor fails to comply with post-market labeling (18). Health Canada also has the discretion to restrict the patient population for which the drug was authorized, restrict distribution, disseminate further educational material for informed use, or enhance post-market surveillance analysis, on a case-by-case basis (15). For example, ponatinib was subject to a controlled distribution program that required prescribers to complete a certification and register prior to prescribing ponatinib (21).

In July 2021, Health Canada released a *Notice of Intent to amend the Food and Drug Regulations and the Medical Devices Regulations to support regulatory agility* (*Notice of Intent*) (16). The amendments are part of a broader modernization initiative toward lifecycle regulation, which trade static, one-time assessments for iterative review throughout a drug's lifespan (22, 23). Amendments under consideration include authorizing the Federal Minister of Health to impose terms and conditions on drug and medical device approvals, based on experience with the NOC/c policy. Terms and conditions will apply predominantly, but not exclusively, to drugs that address a serious or severely debilitating disease or condition and emergencies. Notably, the broader implementation of terms and conditions is not intended to enable drug submissions that do not meet the regulatory requirements. This intention suggests that the flexibility afforded under the current NOC/c policy may not continue, but the lack of detail in the *Notice of Intent* does not enable a fulsome evaluation of the impact of proposed regulations on regulatory approval processes in Canada. It is therefore timely to consider the benefits and limitations of the existing NOC/c process to inform the development of the new agile regulatory framework.

## Analytical Approach

We compiled a comprehensive list of all drugs approved under the NOC/c policy by searching multiple databases, because Health Canada does not maintain a single, up-to-date list of all drugs approved under the NOC/c policy. We searched the publicly available list of drugs on Health Canada's NOC/c webpage (24), the NOC database (25), Health Canada's Drug and Health Product Register (26), Health Canada's Drug and Health Product Submissions Under Review Database (27), and archived versions of Health Canada's NOC/c webpage from the Wayback Machine, a digital archive of internet webpages (28). The Wayback Machine permitted us to include NOC/c information that was previously posted on Health Canada's websites, but has since been deleted or removed. We included all approvals between January 1, 1998 and June 30, 2021.

Health Canada publishes online summaries that describe new drug approvals and new indication approvals. To determine how the eligibility criteria were assessed prior to approval under the NOC/c policy, we searched Health Canada's Drug and Health Product Register to identify published Regulatory Decision Summaries and Summary Basis of Decisions (Regulatory Summaries). We compared the Regulatory Summaries against the eligibility criteria as described in the NOC/c Guidance Document and used the Regulatory Summaries to identify post-market confirmatory trials and the submission status of post-market confirmatory trials. We further reviewed health technology assessments conducted and published by the Canadian Agency for Drugs and Technologies in Health for supplementary information regarding the availability of alternative therapies where available.

We searched the Drugs@FDA Database (29) and Accelerated Approval List (30) to determine the US approval status of drugs approved under the NOC/c policy. Approvals were matched between the US and Canada if the drug was indicated for the same type of cancer and the same line of therapy. We searched Clinicaltrials.gov to identify the status of confirmatory trials for indications with active conditions, because most clinical trials for Canadian regulatory review are registered on this site to meet Health Canada registration requirements. Finally, we reviewed product monographs to confirm the approved indication following submission of post-market confirmatory trials.

## Findings and Discussion: Two Challenges For Conditional Approval Schemes

**Figure 1:**
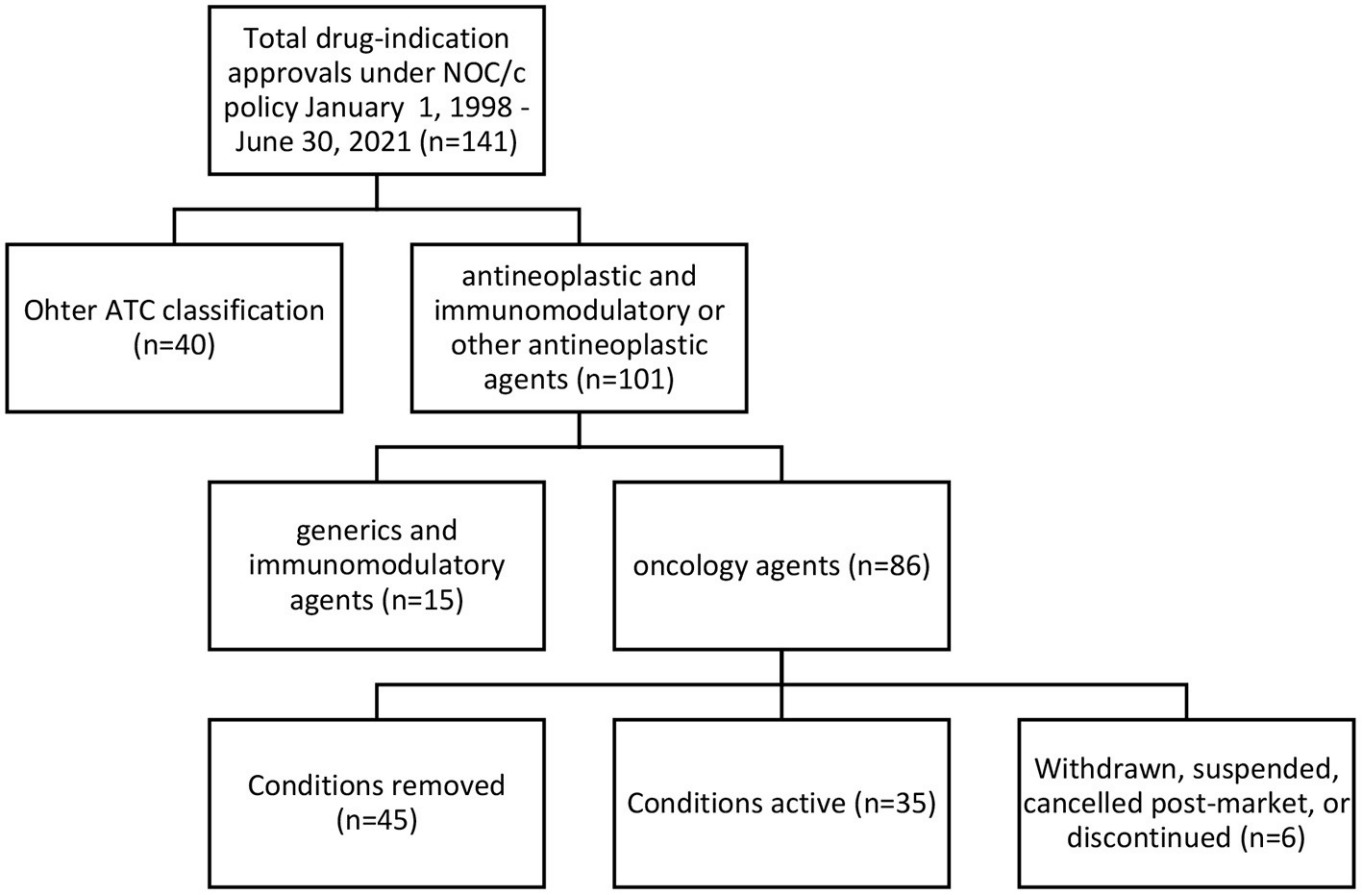
Regulatory status of oncology approvals under NOC/c policy, January 1, 1998–June 30, 2021.

We identified 141 indications approved for 93 drugs (see Supplementary Table 1). Of these, we classified 101 (71% of approvals) as antineoplastic and immunomodulatory agents or other antineoplastic agents according to Anatomical Therapeutic Chemical classification as stated in the Drug Product Database (31). After excluding 12 generics and three immunomodulatory drugs not approved for oncology indications (lenalidomide, eculizumab and ocrelizumab), we included 86 oncology approvals (see Supplementary Table 2) for 58 drugs in our analysis; 14 drugs were approved for two to nine indications. Of the 86 oncology approvals, Health Canada had removed conditions for 45 and transferred their approval to a Notice of Compliance; 35 had active conditions in place; 2 were withdrawn by the manufacturer; 2 were suspended under the *FDR*; 1 was canceled post-market by the manufacturer; and 1 was discontinued by the manufacturer (see Figure 1). 33 approvals had published Regulatory Summaries and were included in our analysis of eligibility criteria.

Our analysis of these oncology approvals focuses on two challenges for conditional approval schemes: first, defining and assessing unmet medical need to determine eligibility for conditional approval, and second, regulatory responsiveness to post-market evidence and regulatory information.

### Unmet Medical Need as an Eligibility Criterion

Eligibility for conditional regulatory pathways is limited to drugs that address unmet medical need. However, there is little consensus on the definition of unmet medical need, and the definition may vary when used by different stakeholders and be context dependent. For example, the definition may differ between the individual patient perspective and population level needs (32) or encompass the availability of an alternative treatment, disease severity or burden, the size of the population, or some combination of these three factors (33).

Currently, the European Union (EU), Canada and the US all have accelerated regulatory pathways that explicitly or implicitly require demonstration of unmet medical need. The regulatory bodies have adopted similar, albeit slightly different definitions of unmet medical need for accelerated or conditional approval pathways. In the EU, unmet medical need is defined in the regulations for conditional authorization of medicinal products as “a condition for which there exists no satisfactory method of diagnosis, prevention or treatment authorized in the Community or, even if such a method exists... the medicinal product concerned will be of major therapeutic advantage to those affected (34).” In the US, FDA defines unmet medical need as “a condition whose treatment or diagnosis is not addressed adequately by available therapy…[a]n unmet medical need includes an immediate need for a defined population (35).” Per this definition, unmet medical need can be met where there is no available therapy, where the only available therapy is approved under the accelerated access program, or where there is available therapy, and the new therapy provides a more favorable benefit risk ratio compared to existing therapy. Although unmet medical need is not an explicit eligibility requirement for the accelerated access pathway in contrast to other expedited pathways, drugs are only eligible for accelerated approval if they provide a meaningful therapeutic benefit over existing treatments (35). In Canada, to be eligible for consideration under the NOC/c policy a drug must be “intended for the treatment, prevention or diagnosis of a serious, life-threatening or severely debilitating disease or condition for which there is no existing therapy…or demonstrates a significant improvement in the benefit/risk profile over alternate available products (15).” Though unmet medical need is not clearly stated as an eligibility requirement, the eligibility criteria are akin to the EU's two-pronged approach.

Unmet medical need as a criterion for accelerated pathways is relevant to oncology because cancers generally meet other potential criteria, such as: serious condition; the multiple paradigms for the categorization of cancers (e.g., histologic and genetic); and treatment often requires multiple sequential or simultaneous combinations of interventions to manage disease progression. However, the interpretation of unmet medical need is inconsistent. One US review of the academic literature identified 237 oncology indications as an unmet medical need, but 55 of these had at least five recommended treatment regimens and a 50% or >5-year survival (36).

In Canada, the NOC/c Policy Guidance Document is inconsistent in defining the two routes for demonstrating eligibility based on unmet medical need—“no existing therapy” and “improvement over existing therapy.” For example, in some parts, “no existing therapy” is defined as “for which no drug is presently marketed in Canada” (ss 1.3, 2.1.3) suggesting the absence of other drugs approved by Health Canada for the indication. Later in the document, the “no existing therapy” criterion is described as available where there is an unmet medical need (s 2.1.2), where an off-label indication is supported by substantial and compelling well-documented evidence (s 1.5), or where no existing therapy possesses a similar therapeutic profile (s 2.1).

Similarly, assessing “improvement over existing therapies” is described using different language in the Guidance Document, making it difficult to discern a clear standard for eligibility. It is unclear what quantum of improvement must be demonstrated to establish a satisfactory improvement in benefit-risk profile. One part of the Guidance Document specifies that there must be an overall improvement in benefit-risk profile, such that an increase in both benefit and risk may still have an overall improvement (s 1.3). Elsewhere, it is stated that a significant improvement or a substantial improvement is required (ss 2.1.2, 2.1.4). Neither substantial nor significant is defined. Additionally, the list of factors that can be used to evaluate the benefit-risk profile of the drug includes “favorable effect on a serious symptom or manifestation of the condition for which there is no existing therapy” (s 2.1.4). It is unclear how this is different from demonstrating that there is no existing therapy, conflating the two eligibility routes.

**Figure 2:**
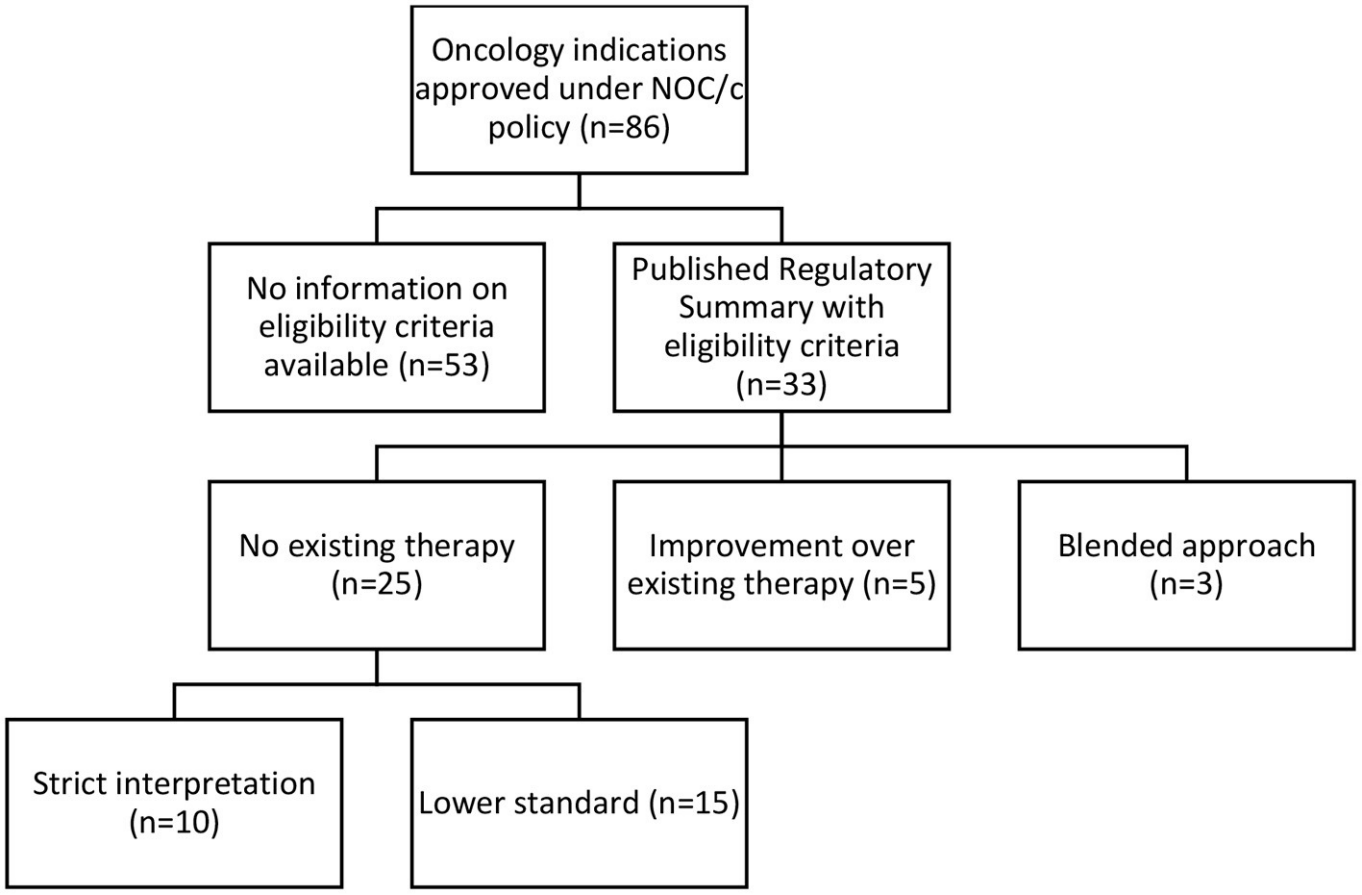
Categorization of NOC/c eligibility criteria.

Our analysis of the 33 available Regulatory Summaries for NOC/c oncology drug approvals in Canada based on “unmet medical need” as a criterion reflected the inconsistencies in the Guidance Document, and it was unclear if unmet medical need is a binary question, a scale, or has different thresholds in different contexts. Twenty-five Regulatory Summaries indicated “no existing therapy” as the basis of the decision, 5 clearly stated that they were an “improvement over existing therapy,” and 3 used a blended approach of these two standards (see Figure 2; Supplementary Table 3).

#### No Existing Therapy Standard

Based on a textual review of the regulatory summaries, Health Canada appeared to apply either a strict interpretation (*n* = 10) or a flexible interpretation (*n* = 15) when assessing the “no existing therapy” criterion. In ten Regulatory Summaries, Health Canada strictly interpreted “no existing therapy” to mean no drug marketed in Canada. Statements supporting this interpretive standard in the Regulatory Summaries included: “no approved treatment,” “no treatment available,” “no authorized agents available,” and other similar variations. We also included one summary in this category that used the term “no effective treatment options.” Though the term “no effective treatment options” suggests blending of the two eligibility routes, because it implies evaluation based on superiority over other treatments, we interpreted it to mean that no marketed drug was available, but perhaps salvage or palliative treatments were being utilized. When we compared these approvals against the Federal oncology-specific health technology assessment process, the pan Canadian Oncology Drug Review recommendations, 7 of these 10 indications had other drugs marketed or treatments available in Canada at the time the health technology assessment was published.

In 15 of the Regulatory Summaries, Health Canada appeared to use a more flexible standard for “no existing therapy,” defined as an indication for which there are few, limited, or a lack of treatments, or where treatments exist but are not effective, proven, or satisfactory. Health Canada provided no reasoning to differentiate these indications from the 10 that were strictly interpreted; it did not use consistent language to describe this flexible standard, stating, “no real treatment options,” “limited treatment options,” that it provided an “alternate treatment option,” or that it addressed “an unmet need.” The flexible standard was justified by subgroups of patients who do not respond to, or poorly tolerate, existing therapies.

It is unclear why sponsors of these 15 drugs were not required to demonstrate an improvement in benefit-risk profile over existing therapies. For example, blinatumomab was approved under the NOC/c policy in 2015 for Philadelphia chromosome-negative relapsed or refractory B precursor acute lymphoblastic leukemia. This approval was justified based on limited existing treatment options for patients who relapse after first line therapy, including aggressive chemotherapy. It met the eligibility criterion by demonstrating a promising clinical benefit for patients who are unresponsive to or unable to tolerate existing therapies. By admitting that there are existing therapies, Health Canada should have required the sponsor to demonstrate an improvement in benefit-risk profile. However, the pivotal trial (NCT01466179) was single arm, making it is unlikely to meet this evidentiary threshold.

One approval in this category for cemipilimab, a programmed cell death 1 (PD-1) immune checkpoint inhibitor, addresses an unmet medical need for an unresponsive subset of patients, reflective of complexities common to oncology treatment. As novel oncology therapies increasingly trend toward genetic based indications, it will be necessary to consider how to define comparator “existing therapies” for subpopulations defined by the sequencing of their biomarkers, particularly in the absence of existing targeted therapies. This consideration is a precondition for a submission using accelerated pathways, including conditional approval.

Many of the approvals in the category were for patient subpopulations that progressed on existing therapies. For example, ceritinib was approved for patients with ALK positive (ALK+) locally advanced or metastatic non-small cell lung cancer (NSCLC) whose disease progressed on crizotinib. The Regulatory Summary defined this as an unmet medical need, categorizing populations with disease progression as a disease “for which no drug is presently marketed (37).” In another example, bosutinib, approved for Philadelphia-chromosome positive chronic myelogenous leukemia (CML) was deemed eligible for “fulfill[ing] an unmet medical need as an alternate treatment option for CML patients who do not respond to or who are intolerant of currently marketed [treatments] in Canada (38).” Defining unmet need in refractory or relapsed settings is another consideration specific to oncology indications and treatment paradigms to determine whether, and how, such circumstances fit into conditional approval regimes.

#### Improvement Over Existing Therapy Standard

The five Regulatory Summaries clearly based on a demonstration of improvement in benefit-risk profile compared to existing treatment used inconsistent language to describe the threshold and quantum of eligibility. Demonstrating improvement compared to existing treatment was defined in some cases as an overall improvement in benefit-risk profile and in others as a significant or considerable improvement in overall benefit-risk profile. The Guidance Document states that to be eligible, the therapy should “provide a statistically significant and clinically relevant improvement in benefit/risk profile, over existing therapies.” (s 2.1.4). However, the difference between an overall, substantial, and significant improvement was not provided in the Guidance Document, and the Regulatory Summaries do not provide definitions or explanations of the thresholds used. Additionally, there is little information on how benefit and risk and quantified, weighed, and compared.

Three of the drugs in this category were approved for second- or third- line therapy for ALK+ NSCLC. This cohort of NOC/c's granted for ALK inhibitors for NSCLC demonstrate a lack of clarity regarding the quantum of improvement over existing therapy required to justify conditional approval, as well as how improvement over existing therapy is determined, particularly in the absence of comparative trials:

1. Alectinib was approved in 2016 as second line therapy for ALK+ NSCLC. Approval under the NOC/c policy was justified because the indication was not adequately managed by available therapies, and it demonstrated the potential to improve the overall benefit-risk profile over existing therapies. The approval was based on two Phase I/II clinical studies (NCT01871805; NCT01801111) without comparator arms, and there was no explanation how it was determined that alectinib was superior to existing therapies. However, in the clinical overview submitted to support its approval, alectinib was differentiated from previously approved therapies, including crizotonib and ceritinib because of its central nervous system activity (39).
2. Brigatinib was approved in 2018 under the NOC/c policy as second line therapy for ALK+ NSCLC on the basis that it was a substantial improvement over existing treatments. The approval of brigatinib was based on two Phase I/II pivotal trials (NCT0144946; NCT02094573) without comparator arms. The improvement over existing therapies was based on the ability of brigatinib to access the central nervous system, and not an objective measure of its improvement over existing therapy.
3. Lorlatinib was approved in 2019 as third-line therapy for ALK+ NSCLC based on its potential to provide an improvement in the benefit-risk profile over existing therapies. Similarly, lorlatinib's potential was based on a phase I/II pivotal trial (NCT01970865) that did not include a comparator arm, and the approval was justified by the lack of third-line therapy for ALK+ NSCLC.

Additionally, alectinib (in 2018) and brigatinib (in 2021) were both approved unconditionally in the first-line setting before the NOC/c approvals in the second-line setting were transferred to an NOC. In both cases, the confirmatory trials required as conditions of the second-line therapy NOC/c were used to support the NOC approval of the drugs in the first-line setting. For alectinib, the confirmatory trials required included two Phase I/II trials in the second line setting (NCT01871805; NCT01801111), and a Phase III trial in the first line setting (NCT02075840). For brigatinib, the only post-market confirmatory trial was a Phase III trial in the first line setting (NCT02737501). This suggests that the confirmatory trials were not designed primarily or solely to address uncertainties in the conditionally approved second-line setting. Instead, the sponsors benefited from 2 to 3 years of extra time on market under patent protection based on Phase I/II clinical trials by identifying an “unmet medical need.” Alectinib had the conditions removed for the second-line indication shortly after the first-line indication was approved, but because Health Canada does not publish Regulatory Summaries when conditions are removed, it is not transparent whether other clinical information in the second-line setting was considered. The FDA has commented on this issue, stating that post-market confirmatory trials are often conducted in earlier-line settings because of enrollment challenges and lack of equipoise when approved therapies are tested in randomized trials (40).

#### Blended Standard

Our category of a “blended” approach for three drugs (**Table 3**) was based on language in the Regulatory Summaries indicative of language asserting both “no existing therapy” and “improvement over existing therapy” as the reason for NOC/c eligibility. The drugs were described as indicated for a disease that was not well managed by adequate therapies in Canada. This language is similar to the second eligibility criterion requiring demonstration of improvement in benefit-risk profile over existing therapies but does not require such demonstration. For example, pralatrexate was approved in 2018 for relapsed or refractory peripheral T-cell lymphoma (PTCL). The approval was based on a phase II single arm trial (NCT00364923). Approval under the NOC/c policy was justified by the limited effective treatment options. The Regulatory Summaries identified three treatment protocols available for the indication but noted limitations in their use in PTCL. It is unclear what the difference is between this circumstance and those in the previous categories that require demonstration of an improvement in benefit-risk profile.

Additionally, there is no clear guidance about when the determination of “available therapies” should take place or be finalized. Most Regulatory Summaries in our analysis contain broad statements about the lack of available treatments, while others explicitly state the lack of available therapies at a specific point in the review process. For example, durvalumab was approved in 2017 as second-line therapy for metastatic urothelial carcinoma. In the Regulatory Summary, eligibility for NOC/c approval was justified because there were no authorized agents available at the time that advanced consideration was granted in December 2016. Prior to durvalumab's conditional approval in November 2017, atezolizumab was approved as second line therapy for urothelial carcinoma under the NOC/c policy in April 2017. The approval of another therapy for the same indication should have required that durvalumab meet the alternative threshold of significant improvement over existing therapies, but instead, the eligibility criterion defined a time point that based the decision on the lack of available therapy at the time of submission. To preserve the integrity of conditional approvals, where a new drug approval renders a pending approval no longer eligible under the criterion of “no existing therapy,” the pending approval should be based on demonstration of “improvement over existing therapies” or be subject to the standard NOC regulatory approval requirements. Alternatively, Health Canada could follow the lead of FDA, which has stated that drugs approved under AA are not considered available for regulatory purposes (40).

In summary, based on the reviewed examples, Health Canada exercises significant flexibility when determining NOC/c eligibility. The NOC/c pathway was designed to provide early approval for drugs that are effective against serious, life-threatening, or disabling diseases that have no existing therapy or drugs that show a significant improvement in their benefit/risk profile compared to existing therapies, for diseases that are not adequately managed by those existing therapies. By using a flexible standard or a blended standard, the NOC/c pathway is allowing drugs that, according to a strict interpretation of the stated criteria, would not be eligible for NOC/c consideration to enter the market early based on unconfirmed clinical effectiveness. While hard definitions may be challenging, and even undesirable to elucidate, particularly relating to the quantum of improvement necessary, at the very least a definition of what qualifies as an existing therapy should be determined and applied.

### Regulatory Responsiveness to Post-market Evidence

A second feature of a conditional drug approval pathway is the need for regulators to respond to post-market evidence that addresses clinical uncertainties at the time of approval. Such new and emerging evidence should inform the ongoing regulatory status of the drug by: confirming the clinical benefit of the drug, changing the approved indication, or withdrawing it from the market. In Canada, drugs that confirm clinical benefit or satisfy other conditions may be transferred from an NOC/c to an NOC approval. However, post approval clinical trials do not necessarily accumulate under existing regulatory requirements, exposing gaps in the current regulatory structure as regulators move toward lifecycle assessment approaches (41). The completion of post-market confirmatory trials is a well-documented challenge, including lack of incentives and difficulty enrolling patients in clinical trials for on-market drugs (2, 7, 42–45).

The ability to respond to evidence collected post-market is central to maintaining the integrity of the regulatory system, public trust in regulatory institutions, and protecting patients from unsafe or ineffective drugs. Without appropriate regulatory responsiveness to post-market evidence, conditional approval pathways may instead lower the threshold for market access. In this section, we analyse regulatory responsiveness to post-market evidence for oncology drugs approved under Canada's NOC/c policy. We assess the regulatory system's ability to collect and use post-market evidence to inform regulatory status and product labels in three ways. We analyzed:

1. Matching approvals under the NOC/c policy and the US AA pathway to identify approvals that have been withdrawn or transferred to a full approval in the US, but remain on market or conditionally approved in Canada;
2. NOC/c approvals with post-market confirmatory trials that are listed as complete on clinicaltrials.gov but remain conditionally approved in Canada; and
3. Labeling changes at the time of transfer to full approval based on the findings of confirmatory trials.

In the absence of greater transparency regarding Health Canada's receipt and review of post-market clinical information, these analyses provide insight into Canada's current capacity to implement a lifecycle regulatory approach in which evidence collection is iterative and informs the regulatory status of the drug on an ongoing basis.

#### Comparative Regulatory Status in US and Canada

**Figure 3:**
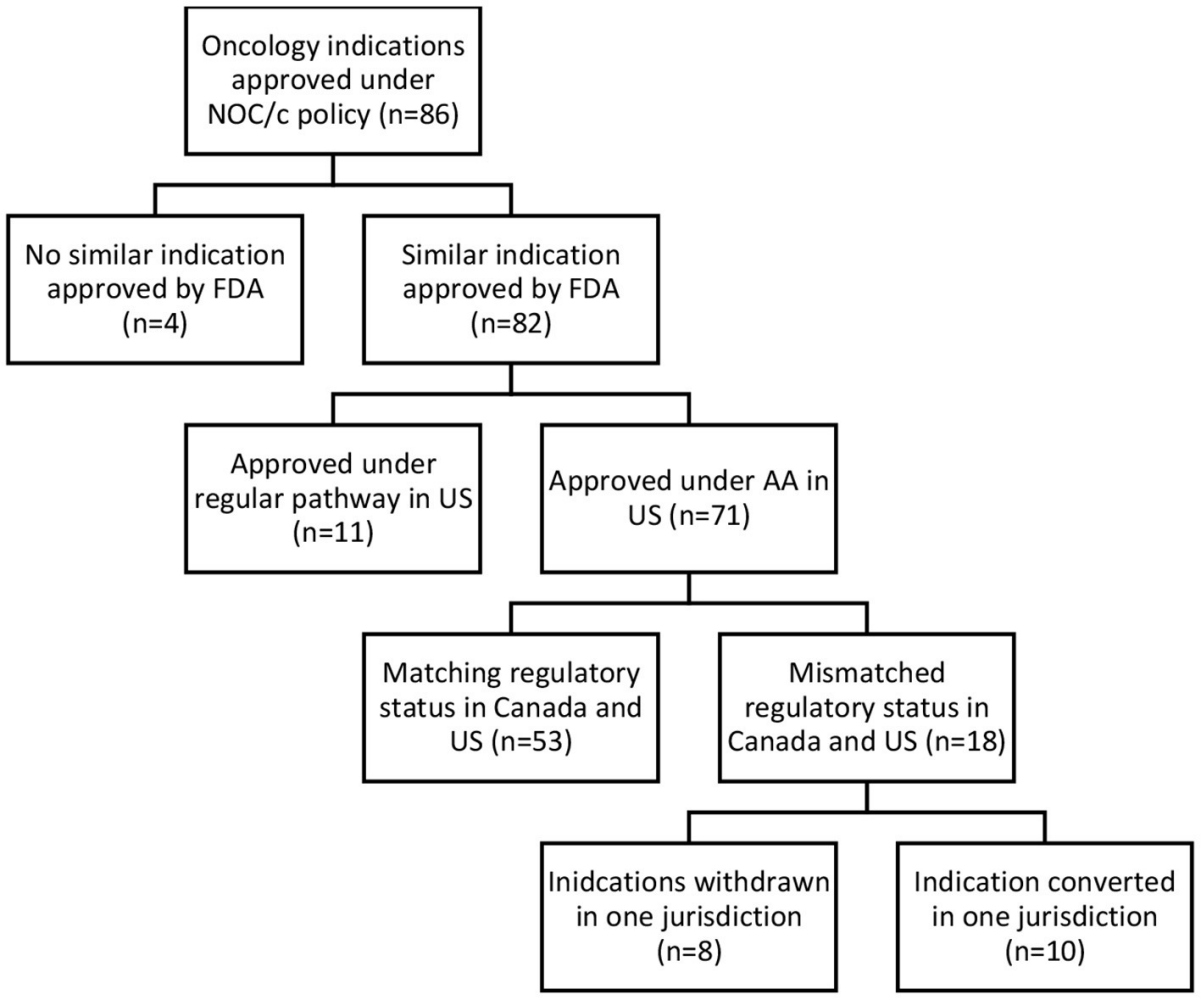
Comparative regulatory status, Canada-United States.

Accelerated and conditional regulatory pathways require the ability to collect, assess, and respond to post-market evidence to ensure regulatory decisions reflect the most up-to-date clinical evidence. To assess regulatory responsiveness, the comparative regulatory status in the US and Canada, status of post-market clinical trials, and post-market label changes were analyzed. Of the 86 oncology drugs approved under the NOC/policy, 82 similar indications were approved by the FDA; 71 under the AA pathway, and 11 *via* regular approval. Of the 71 approved under both the NOC/c and AA pathways, 53 had matching regulatory status (still conditionally approved in both jurisdictions, transferred to regular approval in both jurisdictions, or withdrawn post-market in both jurisdictions). The remaining 18 indications had a different regulatory status in each jurisdiction, including drugs withdrawn post-market in one jurisdiction, but not the other, and indications that have been transferred to a full approval in only one jurisdiction (see Figure 3).

Eight indications approved under the NOC/c policy and AA have been withdrawn post-market in one jurisdiction but not the other (Table 1). Three drugs currently approved in Canada under the NOC/c policy have either been voluntarily withdrawn or recommended for withdrawal in the US by the FDA Oncologic Drugs Advisory Committee (ODAC), because post-market clinical trials failed to confirm benefit in the indication. Nivolumab was approved under the NOC/c policy in 2018 for hepatocellular carcinoma. This indication was slated for review by FDA because of low response rates, changing treatment landscape, failure of the confirmatory trial to confirm benefit, negative results from a monotherapy trial in first line setting, and the potential for alternative trials. ODAC voted 5–4 to remove the indication, largely because the combination indication will remain available, which has superior results (46). Following this, Bristol Myers Squibb voluntarily withdrew the indication in the US (47), but the drug/indication remains conditionally approved in Canada. Similarly, durvalumab and atezolizumab were approved in 2016 and 2017, respectively, in both Canada and the US as second-line therapy for urothelial carcinoma. Both approvals were voluntarily withdrawn in the US by the sponsors after the confirmatory trials did not meet their primary endpoints (48, 49). The Canadian product monographs for durvalumab and atezolizumab, which have both been revised since the withdrawals in the US, indicate that the urothelial carcinoma indications are still approved conditionally in Canada (50).

**Table 1:** NOC/c and AA approvals, withdrawn in one jurisdiction.

**Drug**	**NOC/c approval date**	**Indication**	**NOC/c status (8/30/2021)**	**US FDA Accelerated Approval Date**	**AA Status (8/30/2021)**
Durvalumab for injection	11/3/2017	Locally advanced or metastatic urothelial carcinoma (second line)	Active	5/1/2017	Voluntarily withdrawn
Nivolumab	3/23/2018	Advanced or metastatic hepatocellular carcinoma (second line)	Active	9/22/2017	Voluntarily withdrawn
Atezolizumab	4/12/2017	Urothelial carcinoma (second line)	Active	5/18/2016	Voluntarily withdrawn
Ofatumumab	3/9/2012	Chronic lymphocytic leukemia refractory to fludarabine and alemtuzumab	Canceled post-market	10/26/2009	Converted 4/17/2014
Bicalutamide	11/25/2002	Localized (T1-T2) prostate cancer	Suspended 8/13/2003	10/4/1995	Converted 12/12/1997
Gefitinib	12/17/2003	Locally advanced or metastatic NSCLC (third line)	Transferred	5/5/2003	Withdrawn 4/25/2012
Pembrolizumab	9/8/2017	Refractory or relapsed classical hodgkin lymphoma	Withdrawn 02/03/2021	3/14/2017	Converted 10/14/2020
Bevacizumab	3/24/2010	GBM after relapse or disease progression	Withdrawn 5/23/2018	5/5/2009	Converted 12/5/2017

*ALK+, anaplastic lymphoma kinase positive; ALL, acute lymphocytic leukemia; cHL, classical Hodgkin's lymphoma; CLL, chronic lymphocytic leukemia; GBM, glioblastoma multiforme; NSCLC, non-small cell lung cancer; Ph-, Philadelphia chromosome negative*.

Gefitinib provides another example of an indication withdrawn in the US and not in Canada. Approved in 2003 as third line therapy for NSCLC, the confirmatory trial demonstrated no survival advantage. Despite this, Health Canada opted not to revoke market authorization because of lack of alternative treatment options, the safety profile of gefitinib, and the evidence that gefitinib shrinks tumors, which Health Canada indicated may lower symptom burden (51). In 2006, Health Canada restricted the approved indication to patients currently benefiting from gefitinib, or patients whose tumors are EGFR expression status positive or unknown. Patients were also required to enroll in a patient registry (52). Even though the confirmatory trial results were available to Health Canada in 2006, the conditions were not officially removed until 2009, when gefitinib was approved unconditionally as first line therapy for NSCLC with EGFR mutations. A different approach was taken in the US. In 2010, the FDA requested that AstraZeneca voluntarily withdraw gefitinib from the market because the post market trials failed. AstraZeneca refused to voluntarily withdraw the indication, instead asking FDA to withdraw approval, which was finalized on April 5, 2012 (53). Gefitinib wasn't approved as first-line therapy for NSCLC in the US until 2015. There have been conflicting assessments of gefitinib's pathway to the market; some argue it is an example of downfalls of conditional or accelerated approvals, while others suggest that it is indicative of the deficiencies in existing regulatory structures to support and promote precision-based indications (54).

While confirmatory trials are often considered the main source of post-approval information, foreign regulatory actions are another signal. For this reason, the NOC/c Guidance Document requests sponsors to “[n]otify Health Canada within 15 days… when an expert panel or advisor committee has been struck in a foreign jurisdiction to address an issue or when there has been significant regulatory action in another jurisdiction, including…removal of a product from the market.” (15). Additionally, sponsors are requested to submit a report on the issue that prompted the action within 30 days. While it is unclear whether there are any consequences for failing to submit “requested” documents, Health Canada has the authority under the *FDR* to require information from sponsors and stop sale of the drug if the information is not submitted within the agreed upon timeline (18). Despite this, no action has been taken in Canada, or at least made public, for the first three examples of indications recently withdrawn in the US that remain conditionally approved in Canada. The fact that there have been withdrawals of drugs approved under the NOC/c policy and/or the AA pathways is not an indictment of the regulatory approach. In fact, withdrawals demonstrate that the approach is functioning as intended. In this section, we do not seek to assess the appropriateness of withdrawals, but instead to assess whether Health Canada is responsive to regulatory withdrawals in the US.

On the other hand, there were also five indications that had been withdrawn, suspended, or discontinued in Canada and not the US. One of the withdrawals under the NOC/c policy was the voluntary withdrawal of pembrolizumab for classical Hodgkin's lymphoma, which was later resubmitted under a slightly different indication. Other examples of drugs withdrawn in Canada and not the US include bevacizumab and ofatumumab. Bevacizumab was approved in 2009 and 2010 for recurrent glioblastoma under the AA pathway and the NOC/c policy, respectively. In 2017, FDA granted full approval to bevacizumab following the results of a confirmatory trial, even though the trial did not meet its primary endpoint of overall survival. In Canada, the indication was withdrawn in 2018. Unfortunately, there is no publicly available information about the withdrawal. Ofatumumab was approved for chronic lymphocytic leukemia refractory to fludarabine and alemtuzumab under the AA pathway in 2009 and under the NOC/c policy in 2012. The approval was transferred, and the indication expanded in 2014 in the US (55). The indication was also expanded in 2014 in Canada, but the conditions were not removed. The drug was canceled post market by the sponsor for commercial reasons in 2017 in non-US markets, including Canada. The clinical trial that was used to support conversion of the AA and expansion of the indication (NCT00748189) was only one of the post-market commitments outlined in the Qualifying Notice, suggesting that the remaining commitments may have been what enabled the removal of conditions in Canada.

The above examples demonstrate that Health Canada's foreign regulatory notification requirement may not be sufficient to ensure regulatory responsiveness to market withdrawals in the US. While it is not necessary for regulatory decisions to align entirely between jurisdictions, foreign regulatory decisions and recommendations can be important regulatory signals, particularly for conditionally approved drugs. We also found that approvals were transferred in the US prior to transfer in Canada, despite often overlapping post-market requirements. When we compared NOC/c approvals against approval status in the US, we identified six indications that had been transferred to standard approval in the US but remain conditionally approved in Canada (Table 2). For example, brigatinib was approved by FDA in April 2017 as second line therapy for ALK+ metastatic NSCLC. At the time of approval, FDA requested that the manufacturer conduct a randomized controlled trial (RCT) to confirm clinical benefit of the drug in the approved indication. The trial was not identified at the time of approval. The same indication was approved in July 2018 in Canada under the NOC/c policy. At the time of approval, the Qualifying Notice specified that the results from ALTA-1L (NCT02737501) were required to confirm clinical benefit. In May 2020, ARIAD Pharmaceuticals submitted results from ALTA-1L that led to conversion of the AA to a regular approval, and also expanded the approved indication to first line therapy (56). As of October 2021, brigatinib remains conditionally approved in Canada. The Post-Authorization Activity Table, published within the Regulatory Summary, has not been updated since 2018, however, it is unknown whether the results from ALTA-1L have been submitted to Health Canada. Other examples of approvals that have submitted post market confirmatory trials and successfully transferred to regular approval status in the US but not Canada include pembrolizumab for primary mediastinal B-cell lymphoma and classical Hodgkin's lymphoma, ceritinib for NSCLC, and ponatinib for chronic myeloid leukemia and acute lymphoblastic leukemia (both indications are included in one approval).

**Table 2:** NOC/c and AA Approvals, converted to regular approval in US and not in Canada.

**Drug**	**NOC/c approval date**	**Indication**	**NOC/c status (8/30/2021)**	**US FDA accelerated approval date**	**AA status (8/30/2021)**
Pembrolizumab	9/21/2018	relapsed or refractory Primary Mediastinal B-cell Lymphoma	Active	6/13/2018	Converted 10/14/2020
Pembrolizumab	2/5/2021	refractory or relapsed cHL	Active	3/14/2017	Converted 10/14/2020
Ceritinib	3/27/2015	ALK+ NSCLC (second line)	Active	4/29/2014	Converted 5/26/2017
Brigatinib	7/26/2018	ALK+ NSCLC (second line)	Active	4/28/2017	Converted 5/22/2020
Blinatumomab	4/28/2017	pediatric patients with Ph- relapsed or refractory B cell precursor ALL	Active	9/1/2016	Converted 7/11/2017
Ponatinib hydrochloride	4/2/2015	CML or Ph+ ALL	Active	12/14/2012	Converted 11/28/2016

*ALL, acute lymphoblastic leukemia; ALK+, anaplastic lymphoma kinase positive; cHL, classical Hodgkins lymphoma; CML, Chronic myelogenous leukemia; NSCLC, non-small cell lung cancer; Ph+, Philadelphia chromosome positive; Ph-, Philadelphia chromosome negative*.

It is unclear why these indications have not been transferred in Canada. While speculative, one of the concerns with the NOC/c policy is the lack of incentive for sponsors to make submissions regarding post-market confirmatory trials. For example, ceritinib and ponatinib are already funded in several provinces so it is certainly feasible that there is little to gain from making the necessary submissions to transfer the approval and officially remove the conditions (57, 58). Indeed, if the results of the confirmatory trial are underwhelming or negative, and could result in withdrawal, limiting the indication, or lower utilization rates (and therefore lower profits), postponing submission may be in the best financial interest of the manufacturer. It is also possible that submissions have been made to Health Canada and are still under review.

There are also four indications (for three drugs) that had been transferred in Canada and not in the US (Table 3). In some cases, the discrepancy is related to variations in the approved indication. Idelalisib was approved under AA in 2014 and under the NOC/c policy in 2015 for follicular lymphoma. In the US, idelalsib was also approved for small lymphocytic lymphoma at the same time. The approval was transferred in Canada in 2020, but has not yet been transferred in the US, likely because of the additional indication. In other cases, the reasons behind the discrepancy are less clear. Ibrutinib was approved under the NOC/c policy in 2015 for relapsed or refractory mantle cell lymphoma (MCL). The conditions were removed, and the approval transferred to a NOC in 2017. Ibrutinib was approved under the AA pathway for adult patients with MCL who have received at least one prior therapy in 2018 and has not yet been transferred to a standard approval. Oddly, ibrutinib was approved under AA in 2013 as a first line therapy for MCL. This indication has also not been transferred. The only other example where a drug had conditions removed in Canada but had not yet been transferred in the US is avelumab. Avelumab was approved under the NOC/c policy as second-line therapy for adults with metastatic Merkel cell carcinoma (MCC) in 2017 and first-line therapy for MCC in 2019, also in adults. Both indications had the conditions removed in January 2021. Avelumab was approved as first line therapy for metastatic MCC in adult and pediatric populations in 2017 and has not yet been transferred. It is presumed the difference in conversion date is because the Canadian approval was in the adult population only, while the FDA approved the indication in children and adults.

**Table 3:** NOC/c and AA Approvals, conditions removed in Canada and not US.

**Drug**	**NOC/c approval date**	**Indication**	**NOC/c status (8/30/2021)**	**US FDA accelerated approval date**	**AA status (8/30/2021)**
Idelalisib	3/27/2015	Follicular lymphoma (third line)	Transferred	7/23/2014	Not yet converted
Avelumab	12/17/2017	Metastatic Merkel cell carcinoma (second line)	Transferred	3/23/2017	Not yet converted
Avelumab	11/5/2019	Metastatic merkel cell carcinoma (first line)	Transferred	3/23/2017	Not yet converted
Ibrutinib	7/28/2015	Relapsed or refractory mantle cell lymphoma	Transferred	2/16/2018	Not yet converted

#### Status of Post-market Clinical Trials

Completion of post-market confirmatory trials is a well-documented concern associated with conditional drug approvals (2, 7, 42–45). Conversely, concerns exist that drugs approved under the NOC/c policy with completed confirmatory trials have not been submitted or assessed by Health Canada in a timely manner. Of the 35 oncology drugs with active conditions, trial identification information was available for 24. Of the 24, eight had at least one post-market confirmatory trial that was listed as complete, yet the indication remained conditionally approved. Due to lagging information available about post-market submissions, it is unclear whether these results have been submitted to or reviewed by Health Canada. We are therefore not able to discriminate between these examples demonstrating a lack of incentive for sponsors to submit results once they are available or a lag in regulatory review.

#### Label Changes Following Submission of Confirmatory Trials

The previous two categories assessed responsiveness to post-market evidence by asking whether the existence of a regulatory decision or confirmatory trial results resulted in any regulatory action by Health Canada. Another concern is the quality of responsiveness when post-market evidence is completed, submitted, and integrated into the drug's regulatory status or label. When Health Canada reviews submissions of confirmatory trials, the process for assessing whether the results are sufficient to support removal of the conditions is not known. In fact, the threshold communicated to sponsors to remove conditions is inconsistent. Of the oncology indications approved under the NOC/c policy, 25 Qualifying Notices included language about the potential for the indication to be withdrawn based on the results of the confirmatory trial. In some cases, the Qualifying Notice specified the circumstances in which the indication can be withdrawn, but withdrawal language is used inconsistently. For example, Qualifying Notices may indicate that the indication can be withdrawn in one or more of the following circumstances: if the studies are unsuccessful, if the study fails to confirm a significant improvement in a clinical endpoint, if the results do not demonstrate an improvement in efficacy, and/or if the results fail to demonstrate a favorable or positive overall risk/benefit assessment. The only public documentation of the submission of confirmatory trials is the Post-Authorization Activity Tables in the Summary Basis of Decisions. However, Summary Basis of Decisions are not available for all drugs, and the Post-Authorization Activity Tables are often two years, or more, out of date. Even when these document the submission of confirmatory trials, the record only states that the submission was sufficient to support removal of conditions, providing little insight as to how Health Canada reviews confirmatory trials, what standards it applies and its decision-making process.

In the absence of more detailed regulatory documentation, to assess Health Canada's responsiveness to confirmatory trial results, we reviewed approved indications whose conditions had been removed (*n* = 45) to assess the impact the of confirmatory trials results. This review revealed the addition of indication “caveats,” which we define as an addition to an existing indication in the product label that modifies its meaning, but not a substantive change to the indication itself. We identified 23 approvals that had caveats included in the indication following removal of conditions (Table 4). Here we review a subset of 8 transfers with caveats for aromatase inhibitors [letrozole (approved for two indications), anastrozole, and exemestane]; second-generation tyrosine kinase inhibitors (dasatinib, nilotinib, bosutinib); and venetoclax. We discuss each in turn.

**Table 4:** Indication caveats in product monographs.

**Drug**	**NOC/c approval date**	**Initially approved indication**	**Caveat in product monograph**
Imatinib	9/20/2001	Adult patients with Ph+ CML in blast, accelerated, or chronic phase (after failure of interferon-ax therapy)	Clinical effectiveness in Philadelphia chromosome-positive chronic myeloid leukemia in blast crisis, accelerated phase or chronic phase (after failure of interferon-alpha therapy) was based on hematologic and cytogenetic response rates (surrogate endpoints), which have shown to be sustained for at least two years
Imatinib	10/8/2003	Adult patients with newly diagnosed Ph+ CML	Clinical effectiveness in newly diagnosed CML was based on progression-free survival, hematologic and cytogenetic response rates (surrogate endpoints) that are reasonably likely to predict clinical benefit in a long-term randomized controlled study
Anastrozole	6/30/2004	Adjuvant treatment of postmenopausal women with hormone receptor positive early breast cancer	Approval is based on superior disease-free survival for ARIMIDEX in comparison to tamoxifen. However, overall survival was not significantly different between the two treatments
Letrozole	4/1/2005	Extended adjuvant treatment of early breast cancer in post-menopausal women who have received prior standard adjuvant tamoxifen therapy	Clinical effectiveness is based on superior Disease-Free Survival (DFS) compared to placebo in the overall study population, at a median follow-up of 28 months. However, overall survival was not significantly different between the two treatments for the overall population and an increase in deaths was seen in node-negative patients in the FEMARA arm vs. the placebo arm
Exemestane	5/12/2006	Adjuvant treatment of early breast cancer	Approval is based on improved disease-free survival for sequential AROMASIN in comparison to continuous tamoxifen. However, overall survival was not significantly different between the two treatments
Sorafenib	7/28/2006	Treatment of locally advanced/metastatic renal cell carcinoma in patients who failed prior cytokine therapy or are considered unsuitable for such therapy	Approval of NEXAVAR for locally advanced/metastatic Renal Cell (clear cell) Carcinoma (RCC) is based on progression-free survival (PFS) in low and intermediate risk (MSKCC prognostic criteria) patients without brain metastasis. Prolongation of overall survival has not been established for NEXAVAR in RCC. The quality of life was not significantly different in the pivotal clinical trial comparing NEXAVAR to placebo
Sunitinab	8/17/2006	Treatment of metastatic renal cell carcinoma of clear cell histology after failure of cytokine-based therapy or in patients who are considered likely to be intolerant of such therapy	Approval for MRCC is based on statistically significant progression free survival in patients with good performance status (ECOG 0-1). There was a trend for overall survival advantage
Letrozole	10/6/2006	For the adjuvant treatment of post-menopausal women with hormone receptor positive early breast cancer	Clinical effectiveness is based on superior Disease-Free Survival (DFS) compared to tamoxifen. Overall survival was not significantly different between the two treatments
Docetaxel	12/14/2006	Adjuvant treatment of patients with operable node-positive breast cancer, in combination with doxorubicin and cyclophosphamide	The effectiveness of TAXOTERE in combination with doxorubicin and cyclophosphamide (TAC) is based on improved disease free survival and overall survival in comparison to the combination of fluorouracil, doxorubicin and cyclophosphamide (FAC). However, the positive benefit for TAC in patients with 4+ nodes was not fully demonstrated since the differences in disease-free survival (DFS) and overall survival (OS) between TAC and FAC were not statistically significant in the 4+ nodes stratum
Dasatinib	3/26/2007	Treatment of adults with chronic, accelerated or blast phase CML with resistance or intolerance to prior therapy including imatinib mesylate	Clinical effectiveness of SPRYCEL in CML is based on the rates of hematologic and cytogenetic responses in clinical trials with a minimum of 24 months of follow-up
Imatinib	5/24/2007	Treatment of pediatric patients with newly diagnosed Ph+ CML in chronic phase	Clinical effectiveness in newly diagnosed CML, was based on hematologic and cytogenetic response rates (surrogate endpoints) in a short-term uncontrolled study in which the majority of patients withdrew from protocol therapy to undergo hematopoietic stem cell transplantation
Sunitinib	5/1/2008	Treatment of metastatic renal cell carcinoma of clear cell histology	Approval for MRCC is based on statistically significant progression free survival in patients with good performance status (ECOG 0-1). There was a trend for overall survival advantage
Nilotinib	9/9/2008	Accelerated phase Ph+ CML in adult patients resistant to or intolerant of at least one prior therapy including imatinib	Clinical effectiveness of TASIGNA^®^ in imatinib-resistant or -intolerant Ph+ CML-AP was based on the confirmed hematologic response rates and the unconfirmed major cytogenetic response rates
Nilotinib	7/22/2010	Treatment of chronic phase Ph+ CML in adult patients resistant to or intolerant of at least one prior therapy including imatinib	Clinical effectiveness of TASIGNA^®^ in imatinib-resistant or -intolerant Ph+ CML-CP was based on the unconfirmed major cytogenetic and complete hematologic response rates
Nilotinib	6/23/2011	Treatment of adult patients with newly diagnosed Ph+ CML in chronic phase	Clinical effectiveness of TASIGNA^®^ in newly diagnosed Ph+ CML-CP is based on major molecular response rate at 12 months and complete cytogenetic response rate by 12 months. As of the 60 month cut off date, no overall survival benefit has been demonstrated
Everolimus	6/30/2011	For the treatment of patients of 3 years of age or older with subependymal giant cell astrocytoma associated with tuberous sclerosis complex that have demonstrated serial growth who are not candidates for surgical resection and for whom immediate surgical intervention is not required	The effectiveness of AFINITOR is based on an analysis of change in SEGA volume. Prescribers should take into consideration that surgical resection can be curative, while treatment with AFINITOR has been shown only to reduce the SEGA volume.
Everolimus	1/25/2013	Adult patients with renal angiomyolipoma associated with tuberous sclerosis complex who do not require immediate surgery	The effectiveness of AFINITOR in the treatment of renal angiomyolipoma is based on an analysis of objective responses in patients treated for a median of 8.3 months in the pivotal phase III placebo-controlled trial
Brentuximab vedotin	2/1/2013	Patients with Hodgkin lymphoma after failure of ASCT or after failure of at least two multi-agent chemotherapy regimens in patients who are not ASCT candidates;	Clinical effectiveness in relapsed or refractory HL was based on promising response rates demonstrated in single-arm trials (see CLINICAL TRIALS). No data demonstrate increased survival with ADCETRIS
Osimertinib	7/5/2016	Patients with locally advanced or metastatic EGFR T790M mutation-positive NSCLC who have progressed on or after EGFR TKI therapy. Validated test is required to identify EGFR T790M mutation-positive status prior to treatment	Marketing authorization was based on results from a randomized Phase III trial (AURA3) demonstrating that TAGRISSO is superior to chemotherapy in prolonging progression-free survival (PFS) as assessed by investigator using RECIST v1.1.
Alectinib	9/29/2016	Monotherapy for the treatment of patients with ALK-positive locally advanced or metastatic NSCLC who have progressed or are intolerant to crizotinib	Marketing authorization of ALENCENSARO for the latter indication is primarily based on tumor objective response rate and duration of response; no overall survival benefit has been demonstrated
Venetoclax	9/30/2016	Monotherapy for the treatment of patients with CLL with 17p deletion who have received at least one prior therapy or patients with CLL without 17p deletion who have received at least one prior therapy and for whom there are no other available treatment options	Clinical effectiveness of VENCLEXTA as monotherapy is based on response rate results from single-arm studies
Avelumab	12/17/2017	Treatment of patients with metastatic Merkel cell carcinoma in previously treated adults	Marketing authorization was based on tumor response and durability of response. An improvement in survival or disease-related symptoms has not yet been established
Avelumab	11/5/2019	Treatment of adult patients with metastatic merkel cell carcinoma	Marketing authorization was based on tumor response and durability of response. An improvement in survival or disease-related symptoms has not yet been established

*ASCT, autologous stem cell treatment; CML, Chronic Myelogenous Leukemia; EGFR, IMiD, Immunomodulatory imide drug MRCC, metastatic renal cell carcinoma; NSCLC, non-small cell lung cancer; PFS, progression free survival; Ph+, Philadelphia chromosome positive; PI, proteasome inhibitor; RCC, renal cell carcinoma; TKI, tyrosine kinase inhibitor; EGFR, epidermal growth factor receptor*.

Health Canada approved three aromatase inhibitors *via* the NOC/c pathway between 2004 and 2006 for the adjuvant treatment of postmenopausal women with hormone receptor positive early breast cancer for which the standard of care was tamoxifen. Letrozole was approved separately for adjuvant treatment and extended adjuvant treatment. All four of the NOC/c approvals were based on disease-free survival, a surrogate for overall survival. When the conditions were removed, no significant difference in overall survival compared to tamoxifen or placebo had been demonstrated. Rather than withdraw the indications or request additional confirmatory trials to resolve uncertainties about efficacy, Health Canada added caveats in the product monographs, stating that “overall survival was not significantly different between [placebo and the approved drug] (59–61).”

Health Canada approved three second-generation tyrosine kinase inhibitors through the NOC/c pathway. Dasatinib and nilotinib were approved, in 2007 and 2008, respectively, as second-line therapy for Philadelphia chromosome positive chronic myelogenous leukemia (Ph+ CML). In 2011, both dasatinib and nilotinib were approved as first-line therapy in Ph+ CML in chronic phase. When these four indications were transferred, Health Canada added caveats to the indications, stating that “clinical effectiveness is based on [major molecular response/complete cytogenic response]…[and] no overall survival benefit has been demonstrated (62).” Bosutinib was approved under the NOC/c policy in 2014 as second-line therapy in Ph+ CML, if treatment with imatinib, dasatinib, and nilotinib was not appropriate. Similarly, when bosutinib was transferred, a caveat was included in the product monograph stating that “[m]arket authorization…is based on cytogenetic and hematologic response rates…[o]verall survival benefit has not been demonstrated (63).

Finally, Health Canada approved venetoclax as second-line monotherapy for patients with chronic lymphocytic leukemia with and without 17p deletion in 2016. The approval was based on interim analysis of a Phase II study (NCT01889186) that measured overall response rate. As a condition of approval, four clinical studies were required, but only one was a phase III trial. However, the phase III trial (NCT02005471) was a combination trial, and as a result, there was no phase III trial for venetoclax as monotherapy in any line of therapy at the time it was transferred. When the condition was removed, a caveat was added stating that “clinical effectiveness of VENCLEXTA as monotherapy is based on response rate results from single arm studies (64).”

These examples provide insight into Health Canada's responsiveness to post-market confirmatory trials and suggest the creation of a new class of drug approvals: drugs that were initially approved conditionally, have been transferred to regular approval, yet never met the standard threshold of substantial evidence of effectiveness demonstrated by two adequate and well controlled clinical trials. These indications no longer carry the mandatory warnings, educational requirements, and consent requirements associated with drugs approved under the NOC/c policy with active conditions that signal to physicians and patients that there are outstanding clinical uncertainties. Instead, there is little to communicate to patients and physicians that these drugs are different from drugs that have been approved and met the regulated standard of substantial evidence of effectiveness. Instead, Health Canada has introduced a quasi-regulatory communication in the form of a caveat to approved indications in product labels in an attempt to mitigate evidentiary uncertainty. The issue of adding caveats is not necessarily confined to the NOC/c policy in Canada; one study in the US found that overall survival data was inconsistently reported in cancer drug labels (65). Our findings confirm that it may be worthwhile to investigate the reporting of overall survival data more broadly for cancer drugs approved in Canada.

### Summary

The lack of definitional clarity for assessing eligibility raises concerns about whether accelerated pathways are being appropriately utilized to expedite patient access to therapies for which no satisfactory treatment options exist. If unmet medical need is not clearly defined and assessed, the tradeoff that forms the basis of the conditional approval pathway may be undermined, and risks becoming a mechanism for drug sponsors to expedite market access. Without clear assessment criteria, drug manufacturers can easily demonstrate that their drug addresses an unmet medical need, rendering the eligibility criterion arbitrary. Granting drugs market approval that have not reached the generally accepted threshold of substantial evidence of safety and efficacy based on an arbitrary determination of whether that drug targets an unmet medical need has the potential to undermine public trust in government institutions and increase the risk to patients, either because of safety signals only detected post-market that would ordinarily have been detected in pre-market clinical trials, or because of patients using drugs that are found out to be ineffective only after already being on market. Accelerated pathways represent important opportunities for new drugs to gain market access that may be precluded under standard regulatory pathways. For example, in the case of drugs for rare diseases and drugs for emergencies, the added certainty associated with waiting for more evidence may be outweighed by the benefit of earlier access. Additionally, in some cases, satisfying the “substantial evidence of clinical effectiveness” requirement may be impractical, or even impossible. However, a clearer definition of unmet medical need will ensure that an accelerated pathway is used to enable patient access to new drugs for serious conditions that address such needs.

Additionally, the examples discussed above demonstrate an underwhelming responsiveness to post-market evidence, in terms of responsiveness to regulatory decisions in the US, confirmatory trials, and the results of confirmatory trials. Regulatory responsiveness is an important trade-off in conditional drug approvals. The promise to withdraw, transfer, or update the indication in a timely manner ensures that patients and physicians have up to date regulatory information to support clinical decision making. Under the proposed approach to impose terms and conditions on drug approvals more broadly, it can be expected that post market confirmatory trials will continue to be relied upon to address uncertainties that exist at the time of approval or that arise post-market. The examples discussed above confirm concerns expressed previously in other jurisdictions that post market confirmatory trials should not be relied upon to address uncertainties under existing regulatory structures (66).

## How Can the Current Noc/C Pathway Inform the Proposed Agile Regulatory Approach?

The new approach to conditional drug approvals in Canada is currently in the consultation stage, with a *Notice of Intent* issued by Health Canada that sets out the contours of a proposed new agile regulatory approach (16). The *Notice of Intent* does not specify whether the NOC/c policy will be replaced by the novel approach, however, it seems redundant to maintain it alongside the proposed agile regulations. Additionally, the *Notice of Intent* claims that the proposed changes will leverage experience with the NOC/c policy to date. A few key features specified in the *Notice of Intent*, when considered in concert with our analysis above, form the basis for our recommendations on the future of conditional regulatory approval approaches in Canada.

First, the new approach seeks to broaden the eligibility criteria, removing the requirement to demonstrate that the drug addresses an unmet medical need, either by targeting an indication for which there is no treatment available, or by demonstrating an improvement over existing therapy. Instead, the *Notice of Intent* specifies that terms and conditions will be used predominantly for: drugs that address a serious, life-threatening, or severely debilitating disease or condition; emergencies; or where there is uncertainty about new drugs that could be addressed by additional clinical trials and real-world experience (16). As a non-exclusive requirement, it can be assumed that broadening the eligibility criteria will result in post-market conditions being used more widely than under the current NOC/c policy.

Broadening the eligibility criteria is concerning for a few reasons. First, it is important to differentiate between imposing terms and conditions at the time of approval compared to imposing terms and conditions in response to safety signals or other new information that becomes available post-approval. In the latter case, there is less concern about the impact of such an approach on the initial approval; reviewers are unlikely to predict or consider the potential for terms and conditions to be added at a later point in time. This tool is a welcome addition in support of a lifecycle regulatory approach by clarifying Health Canada's ability to require the submission of clinical information. However, the power to add terms and conditions at the point of initial market approval based on broad eligibility criteria is concerning because of the impact that terms and conditions may have on the approval decision making process. When reviewers are aware that post-market conditions can or will be imposed on an approval, they may, whether intentionally or not, be more accepting of evidence gaps or rely too heavily on the ability of deferred post-market activities to resolve uncertainties (67). Additionally, research has demonstrated that accelerated approval leads to quicker patient access to drugs, but slower access to crucial clinical information because of the barriers to completing randomized clinical trials once a drug is already on market (68–70). The case studies discussed above confirm that post-market evidence does not necessarily accumulate to inform regulatory status and approved indications. Terms and conditions imposed at the time of initial approval should be limited to narrow circumstances where the benefits of earlier access clearly outweigh the additional risks associated with approving a drug based on less mature evidence.

Broadening eligibility could be justified by less flexible evidence requirements under the proposed approach, which suggests that Health Canada will no longer permit approvals not meeting the regulatory standard of “substantial evidence of clinical effectiveness,” typically interpreted as requiring “at least two adequate and well controlled clinical studies, each convincing on its own to establish effectiveness of the drug (19).” There is reason to be suspect of the feasibility of such an approach. Approvals, particularly for oncology drugs under the NOC/c policy over the last decade, have demonstrated deviations from the standard regulatory threshold (7). Additionally, alternative clinical trial designs, such as basket and umbrella trials are increasingly relied upon for clinical development of oncology drugs targeting rare biomarkers or small patient populations, posing challenges for strict adherence to the requirement for RCTs (71, 72). Much like it is more difficult to withdraw a drug approval than to not approve it in the first place, it can be expected that upholding a higher evidence threshold after decades of permissiveness will face significant pushback from industry.

Whether Health Canada will uphold the standard set out in regulations or continue to exercise flexibility in assessing drug submissions will remain to be seen, however experience in the US may provide some insight. FDA states that submissions for approval under the AA pathway must meet the same statutory standard for effectiveness as drugs approved through regular approval pathways, which requires substantial evidence based on adequate and well controlled clinical trials (73). Despite this requirement, studies have found that this is not rigorously applied. One study found that 14/24 reviewed approvals granted AA were based on non-randomized, non-comparative single-group studies (12). Similar findings have been found for drug approvals more generally; one study found that that the proportion of approvals supported by only single-group pivotal trials has increased from 1995 to 2017 (74). Furthermore, several studies have found that approving drugs with limited clinical evidence, once intended to be the exception has instead become the new norm (66, 75, 76). Notwithstanding a literal reading of the new regulatory approach, it seems unlikely that Canada will stop exercising flexibility to approve drugs with promising evidence and start requiring strict adherence to regulatory requirements requiring substantial evidence of clinical effectiveness. Flexible regulatory approaches remain an important route to market for many types of drugs and conditions, and have become standard internationally.

Under the proposed regulatory system, which would replace the NOC/c policy with the broader ability to impose terms and conditions on drug approvals, demonstrating eligibility criteria will be a much simpler exercise. The *Notice of Intent* states that the use of terms and conditions is intended for drugs that address a serious or severely debilitating disease or emergency circumstances. While this approach will remove the challenges associated with defining and assessing unmet medical need, it also fundamentally changes the balance that conditional approvals seek to strike between earlier access to drugs and mitigating uncertainty. Restricting eligibility to drugs that address an unmet medical need, however it may be defined, limits the privilege of earlier access to circumstances where patients have few or no other treatment options. In such circumstances, patients may be more willing to accept the higher risk associated with the uncertainty of approval based on less evidence. In any case, clear and comprehensive definitions that account for clinical realities should be developed to guide the application of assessment for terms and conditions under the new approach. As we have demonstrated, poorly defined regulatory requirements result in inconsistent and unpredictable application of the current NOC/c Guidance. While it does not appear from the *Notice of Intent* that unmet medical need will continue to be an eligibility requirement under the new approach, if it is, developing clear definitions for when an unmet medical need exists, including how to define the availability of existing therapies, will be needed.

Second, the new approach to terms and conditions on drug authorizations will address some of the major challenges identified with the NOC/c policy associated with completion and submission of clinical trials by enshrining the ability to add terms and conditions on drug approvals in the regulations. Currently, the NOC/c policy is guided only by policy, including the non-legally binding Guidance Document. The power to add terms and conditions to drug approvals is an important step toward adopting a lifecycle approach to the regulation of drugs. Under more traditional regulatory approaches, regulators often had little power to compel drug manufacturers to conduct studies or share information in response to post-market safety or efficacy issues. Coupled with the lack of incentives for drug manufacturers to conduct studies and collect information that could adversely impact their regulatory standing, useful post-market clinical information was not often collected (2, 41, 77–79). Health Canada should be commended for introducing regulations that empower the Minister of Health to collect and respond to post-market evidence on all drug approvals, not just those that are conditionally approved. However, experience with conditional approvals in Canada and abroad indicate that withdrawing or limiting approved indications is much more difficult to do in practice than it is to delay or avoid approving them in the first place (67, 80–82). Even jurisdictions such as the US with the regulatory power to enforce post-market confirmatory trials requirements have faced challenges ensuring their timely completion (2, 45, 80, 83), suggesting that the power to enforce completion and submission of confirmatory trials is not sufficient.

Many have recommended alterations to the mechanisms for assessing the status of post-market conditions to mitigate the delays and maladapted incentives inherent in current approaches in Canada and the US. Recommendations include requiring confirmatory trials to be underway at the time of approval, transparent and strictly enforced deadlines, harsher penalties for non-compliance, and automatic review of conditions to avoid “dangling” approvals, sluggish trials, and to permit for rapidly changing therapeutic landscapes (4, 7, 46, 78). These should be considered in Canada's new regulatory approach. To adopt such mechanisms, Health Canada will need to respond to conditionally approved drugs that are either non-compliant or rendered redundant because of new treatment options. Allowing drugs to remain on market in these circumstances leaves patients and physicians to bear the risks associated with prolonged uncertainty, while drug manufacturers reap the rewards of earlier market access. It is not sufficient to simply include the threat of financial penalties for non-compliance or withdrawal of regulatory approval. Additionally, these regulatory approaches are focused on penalizing the sponsor with little consideration for how to address the concerns and needs of patients and physicians who are using the drugs. It is also important to consider potential regulatory tools that can be adopted to manage rapidly changing therapeutic landscapes, which can render conditional approvals obsolete and further disincentive drug manufacturers to further invest in confirmatory trials.

Our final recommendation applies both to pre-approval eligibility assessments and post-market confirmatory trials. Our analysis confirms that lack of transparency about decision-making processes is a considerable barrier to understanding and evaluating the NOC/c policy (5–7). The *Notice of Intent* does not specifically address transparency measures. To promote accountability, Health Canada will need to ensure transparency of the content and status of conditions, as well as the decision frameworks used both to decide whether to implement conditions, and whether to remove them. Clear decision frameworks are necessary to ensure not only accountability, but to encourage consistent decision making. Increased transparency will also go a long way to increasing consistency in decision making; a challenge we have highlighted in our above analysis that has also been identified in the US (84). The FDA Oncology Center of Excellence recently announced Project Confirm, an initiative to “promote the transparency of outcomes related to Accelerated Approvals for oncology indications (40).” Initiatives such as Project Confirm demonstrate the demand for transparency measures that address oncology-specific concerns.

## Limitations

Information on NOC/c's granted between 1998 and 2004 were not available through the wayback machine, so this analysis likely underestimates the total number of drugs approved under the NOC/c policy, particularly for this time period. In addition, many Health Canada websites and databases are not up to date. There is often a time lag between when drugs are approved and when Regulatory Summaries are published, so these documents are not typically available for several years after approval. There was significant variability in the amount of information available for each drug/indication, depending on what documents were publicly available. In addition, there were often discrepancies in the information provided between sources. Further, Health Canada does not make available information on whether clinical trial results or notice of foreign regulatory decisions have been submitted, so it is possible that some of the information is still under consideration by Health Canada. Our analysis relied upon information presented in publicly available documents to discern Health Canada's decision-making processes, but there may have been additional information reviewed or considered, and the public documents may not fully reflect the decision-making process nor the evidence available to Health Canada. Additionally, our review was limited to drugs approved under the NOC/c policy. We did not review drugs approved under the regular drug approval pathway. As a result, we cannot be certain that our findings are unique to the NOC/c policy.

## Conclusion

Conditional approval pathways have represented a significant path to market for new oncology drugs and indications over the last decade. Conditional approval pathways in Canada and abroad have been subject to ongoing criticism for lack of enforceability and lack of transparency. Here we assessed two components of the NOC/c policy that represent core tradeoffs of conditional approval pathways: limiting eligibility to drugs that address an unmet medical need, and regulatory responsiveness. Experience to date with these components of the policy are relevant for informing the further development of Health Canada's proposed agile regulations for drugs. Our analysis revealed that eligibility criteria are not clearly defined and inconsistently applied under the NOC/c policy, undermining the justification of earlier market access based on less mature evidence. Broadening eligibility for post-market conditions to include drugs for all indications is appropriate when implemented in response to new post-market information. However, broadening eligibility criteria to implement terms and conditions at initial regulatory approval decision is more concerning. The added risk of permitting earlier access to drugs based on immature clinical evidence is typically justified by unmet medical need. Despite an intent not to permit deviation from the regulatory standard of demonstrating clinical effectiveness, there is reason to suspect the feasibility of applying stringent regulatory standards after an extended period of flexibility.

Our analysis also considered regulatory responsiveness, assessed by Health Canada's response to regulatory decisions made in the US, responsiveness to completed confirmatory trials, and indication changes following receipt and review of confirmatory trial results. Across the first two categories, Health Canada's responsiveness is slow. As a result, conditionally approved drugs and indications remain available in Canada after they have been withdrawn in the US or remain conditionally approved after they have been transferred to standard approval in the US and after the results of the confirmatory trials are available. The ability for regulatory status to be updated in response to new information is crucial to uphold the integrity of conditional regulatory approval pathways and to ensure that patients and physicians have the most up to date information available to them. Additionally, there was a small cohort of drugs that had conditions removed and caveats added to the indication, suggesting a new class of approved drugs that have neither met the evidence standards expected of approved drugs (substantial evidence of clinical effectiveness), nor carry the added labeling and warning requirements associated with conditionally approved drugs. Together, these examples suggest that current mechanisms for collecting, assessing, and responding to evidence collected post-market are not sufficient to inform regulatory status and clinical practice.

Experience to date with the NOC/c policy is useful for guiding the further development of the new agile regulatory approach for drugs. While enshrining enforcement mechanisms in regulations is an important amendment to the current approach, further consideration of assessing eligibility and enforcing post-market commitments is needed to encourage appropriate use of post-market terms and conditions.

## Author Contributions

MM and EW collected and analyzed the regulatory and clinical data and information. MM prepared the first draft of the manuscript. TB wrote sections of the manuscript. All authors contributed to manuscript revision, read, and approved the submitted version.

## Funding

The Canadian Network for Learning Healthcare Systems and Cost-effective ‘Omics Innovation is funded by Genome British Columbia/Genome Canada (G05CHS).

## Conflict of Interest

The authors declare that the research was conducted in the absence of any commercial or financial relationships that could be construed as a potential conflict of interest.

## Publisher's Note

All claims expressed in this article are solely those of the authors and do not necessarily represent those of their affiliated organizations, or those of the publisher, the editors and the reviewers. Any product that may be evaluated in this article, or claim that may be made by its manufacturer, is not guaranteed or endorsed by the publisher.
