# Supplementary material for: Conditional Drug Approval as a Path to Market for Oncology Drugs in Canada: Challenges and Recommendations for Assessing Eligibility and Regulatory Responsiveness
Source: Front Med (Lausanne). 2022 Feb 3;8:818647. doi: 10.3389/fmed.2021.818647 (PMC8853442; doi:10.3389/fmed.2021.818647)
Supplement: Supplementary file 1 [file Data_Sheet_1.docx]

Supplementary Material

# Supplementary Table 1: List of all drugs approved under the NOC/c Policy in Canada (January 1, 1998- June, 30, 2021)

| Drug | **Date NOC/c granted** | **Date Conditions Met** | **Indication** |
| --- | --- | --- | --- |
| delavirdine | 7/22/1998 | 7/22/2003 | not specified |
| nevirapine | 9/4/1998 | 9/13/2004 | not specified |
| recombinant factor VII activated | 2/12/1999 | 5/19/2006 | bleeding episodes in patients who have hemophilia A or B with inhibitors to clotting factors VIII or IX |
| alteplase | 2/16/1999 | 1/26/2005 | management of acute ischemic stroke in adults to improve neurological recovery and reduce disability |
| abacavir sulfate | 6/4/1999 | 9/10/2001 | not specified |
| zanamivir | 11/2/1999 | 8/26/2003 | Treatment of influenza in paediatrics 7 years and older |
| riluzole | 8/30/2000 | 11/29/2007 | Amyotrophic lateral sclerosis |
| amprenavir | 3/1/2001 | 7/5/2004 | not specified |
| imatinib | 9/20/2001 | 12/29/2004 | Adult patients with Ph+ CML in blast crisis, accelerated phase or in chronic phase (after failure of interferon-ax therapy). |
| tenofovir disoproxil fumarate | 3/18/2003 | 7/20/2005 | treatment of HIV-1 infection in combination with other antiretroviral agents in patients 18 years and older who have experienced virologic failure on other regimens |
| bicalutamide | 8/13/2003 | 8/13/2003 | for immediate therapy in some patients with localized (T1-T2) prostate cancer who are inappropriate for surgery or radiotherapy |
| imatinib | 10/8/2003 | 6/17/2010 | Adult patients with newly diagnosed Ph+ CML |
| gefitinib | 12/17/2003 | 12/18/2009 | third line treatment of patients with locally advanced or metastatic NSCLC after failure of two prior chemotherapy regimens |
| agalsidase alfa | 2/6/2004 | 6/20/2004 | enzyme replacement therapy for fabry disease |
| celecoxib | 5/20/2004 | 12/17/2004 | treatment/prevention of familial adenomatous polyposis |
| anastrozole | 6/30/2004 | 12/2/2008 | adjuvant treatment of postmenopausal women with hormone receptor positive early breast cancer |
| memantine hydrochloride | 12/8/2004 | 4/21/2011 | treatment of patients with moderate to severe dementia of the Alzheimer’s type |
| bortezomib | 1/27/2005 | 9/11/2007 | treatment of multiple myeloma patients who have relapsed following front-line therapy and refractory to their most recent therapy |
| letrozole | 4/1/2005 | 12/17/2010 | for the extended adjuvant treatment of early breast cancer in post-menopausal women who have received prior standard adjuvant tamoxifen therapy |
| delta-9-tetrahydrocannabinol/cannabidiol | 4/15/2005 | 12/12/2019 | symptomatic relief of neuropathic pain in multiple sclerosis |
| capecitabine | 12/7/2005 | 10/23/2008 | adjuvant treatment of patients with Stage III (Dukes' stage C) colon cancer |
| exemestane | 5/12/2006 | 6/6/2008 | adjuvant treatment of early breast cancer |
| sorafenib tablets | 7/28/2006 | 6/12/2009 | treatment of locally advanced/metastatic renal cell carcinoma in patients who failed prior cytokine therapy or are considered unsuitable for such therapy |
| sunitinab malate | 8/17/2006 | 4/23/2010 | treatment of metastatic renal cell carcinoma of clear cell histology after failure of cytokine-based therapy or in patients who are considered likely to be intolerant of such therapy |
| letrozole | 10/6/2006 | 12/17/2010 | for the adjuvant treatment of post-menopausal women with hormone receptor positive early breast cancer |
| deferasirox | 10/18/2006 | 12/2/2016 | management of chronic iron overload in patients with transfusion dependent anemias in patients 6 years or older or aged 2-5 who cannot be adequately treated with deferoxamine |
| docetaxel | 12/14/2006 | 9/26/2012 | adjuvant treatment of patients with operable node-positive breast cancer, in combination with doxorubicin and cyclophosphamide |
| hepatitis B Immune Globulin | 1/19/2007 | n/a | prevention of hepatitis B recurrence following liver transplantation in adult patients with hepatitis B who have no or low levels of HBV replication |
| levodopa/carbidopa | 3/1/2007 | 3/12/2014 | advanced levodopa-responsive Parkinson's disease who do not have satisfactory control of motor fluctuations and hyper/dyskinesia despite optimized treatment with available combinations of Parkinson’s disease medicinal products |
| dasatinib | 3/26/2007 | 11/19/2009 | treatment of adults with chronic, accelerated or blast phase CML with resistance or intolerance to prior therapy including imatinib mesylate |
| imatinib mesylate | 5/24/2007 | 2/21/2013 | Treatment of pediatric patients with newly diagnosed Ph+ CML in chronic phase |
| delta-9-tetrahydrocannabinol/cannabidiol | 8/1/2007 | 12/12/2019 | adjuvant analgesic treatment in adult patients with advanced cancer who experience moderate to severe pain during the highest tolerated dose of strong opioid therapy for persistent background pain |
| nelarabine | 9/22/2007 | 1/22/2020 | Patients with refractory or recurrent T-ALL or T-lymphoblastic lymphoma |
| nesiritide | 11/8/2007 | 8/1/2012 | treatment of hospitalized patients with acute episodes of heart failure who did not respond to treatment with diuretic drugs for two hours |
| pregabalin | 11/9/2007 | 6/29/2010 | symptomatic relief of neuropathic pain associated with damaged nerves of the CNS including the brain and spinal cord |
| raltegravir potassium | 11/27/2007 | 3/4/2009 | in combination with other antiretroviral agents, is indicated for the treatment of HIV-1 infection in treatment experienced adult patients who have evidence of viral replication and HIV-1 strains resistant to multiple antiretroviral agents. |
| lenalidomide | 1/17/2008 | 6/6/2013 | treatment of patients with transfusion dependent anemia due to low or intermediate 1 risk myelodysplastic syndromes associated with deletion 5q cytogenic abnormality with or without additional cytogenetic abnormalities |
| panitumumab | 4/3/2008 | 2/19/2015 | monotherapy for the treatment of patients with EGFR expressing metastatic colorectal carcinoma with non-mutated (wild type) KRAS after failure of fluoropyrimidine-, oxaliplatin- and irinotecan-containing chemotherapy regimens |
| sunitinab malate | 5/1/2008 | 4/23/2010 | treatment of metastatic renal cell carcinoma of clear cell histology |
| darunavir/TMC114 | 6/18/2008 | 2/11/2009 | co-administered with ritonavir and with other ARV agents for the treatment of HIV infection in treatment-experienced adult patients who have failed prior antiretroviral therapy |
| idebenone | 7/23/2008 | 4/30/2013 | Friedrich's Ataxia |
| nilotinib | 9/9/2008 | 11/30/2011 | accelerated phase Ph+ CML in adult patients resistant to or intolerant of at least one prior therapy including imatinib |
| bevacizumab | 2/6/2009 | 11/25/2011 | used in combination with paclitaxel for treatment of patients with HER2 negative breast cancer and who are ECOG class 0-1 |
| aztreonam for inhalation solution | 9/11/2009 | 5/17/2011 | for the management of cystic fibrosis patients with chronic pulmonary PA infections |
| memantine hydrochloride | 10/29/2009 | 4/21/2011 | not specified |
| memantine hydrochloride | 11/4/2009 | 4/21/2011 | not specified |
| memantine hydrochloride | 11/25/2009 | 4/21/2011 | not specified |
| imatinib mesylate | 12/22/2009 | 11/24/2017 | For the adjuvant treatment of adult patients who are at intermediate to high risk of relapse following complete resection of Kit (CD117) positive GIST |
| bevacizumab | 3/24/2010 | 5/23/2018 | for the treatment of patients with GBM after relapse or disease progression |
| letrozole | 4/7/2010 | 2/14/2013 | (1) adjuvant treatment of postmenopausal women with hormone receptor positive early breast cancer, and  (2) the extended adjuvant treatment of hormone receptor positive early breast cancer in postmenopausal women who have received approximately 5 years of prior standard adjuvant tamoxifen therapy |
| memantine hydrochloride | 4/16/2010 | 12/20/2011 | symptomatic treatment of patients with moderate to severe dementia of the Alzheimer’s type |
| letrozole | 4/28/2010 | 12/2/2011 | (1) adjuvant treatment of postmenopausal women with hormone receptor positive early breast cancer, and  (2) the extended adjuvant treatment of hormone receptor positive early breast cancer in postmenopausal women who have received approximately 5 years of prior standard adjuvant tamoxifen therapy |
| letrozole | 4/28/2010 | 8/16/2011 | (1) adjuvant treatment of postmenopausal women with hormone receptor positive early breast cancer, and  (2) the extended adjuvant treatment of hormone receptor positive early breast cancer in postmenopausal women who have received approximately 5 years of prior standard adjuvant tamoxifen therapy |
| letrozole | 4/28/2010 | 12/10/2013 | not specified |
| letrozole | 4/28/2010 | 6/14/2011 | (1) adjuvant treatment of postmenopausal women with hormone receptor positive early breast cancer, and  (2) the extended adjuvant treatment of hormone receptor positive early breast cancer in postmenopausal women who have received approximately 5 years of prior standard adjuvant tamoxifen therapy |
| letrozole | 4/28/2010 | 6/24/2011 | (1) adjuvant treatment of postmenopausal women with hormone receptor positive early breast cancer, and  (2) the extended adjuvant treatment of hormone receptor positive early breast cancer in postmenopausal women who have received approximately 5 years of prior standard adjuvant tamoxifen therapy |
| letrozole | 4/28/2010 | 6/22/2011 | (1) adjuvant treatment of postmenopausal women with hormone receptor positive early breast cancer, and  (2) the extended adjuvant treatment of hormone receptor positive early breast cancer in postmenopausal women who have received approximately 5 years of prior standard adjuvant tamoxifen therapy |
| memantine hydrochloride | 5/13/2010 | 12/21/2013 | symptomatic treatment of patients with moderate to severe dementia of the Alzheimer’s type |
| nilotinib | 7/22/2010 | 8/18/2011 | treatment of chronic phase Ph+ CML in adult patients resistant to or intolerant of at least one prior therapy including imatinib |
| letrozole | 8/27/2010 | 5/20/2011 | (1) adjuvant treatment of postmenopausal women with hormone receptor positive early breast cancer, and  (2) the extended adjuvant treatment of hormone receptor positive early breast cancer in postmenopausal women who have received approximately 5 years of prior standard adjuvant tamoxifen therapy |
| memantine hydrochloride | 10/5/2010 | 12/21/2011 | symptomatic treatment of patients with moderate to severe dementia of the Alzheimer's type |
| letrozole | 12/9/2010 | 9/6/2013 | (1) adjuvant treatment of postmenopausal women with hormone receptor positive early breast cancer, and  (2) the extended adjuvant treatment of hormone receptor positive early breast cancer in postmenopausal women who have received approximately 5 years of prior standard adjuvant tamoxifen therapy |
| docetaxel | 2/25/2011 | 10/10/2013 | for use in combination with doxorubicin and cyclophosphamide for the adjuvant treatment of patients with operable node positive breast cancer |
| nilotinib | 6/23/2011 | 8/19/2015 | treatment of adult patients with newly diagnosed Ph+ CML in chronic phase |
| everolimus | 6/30/2011 | 4/29/2016 | for the treatment of patients of 3 years of age or older with subependymal giant cell astrocytoma associated with tuberous sclerosis complex that have demonstrated serial growth who are not candidates for surgical resection and for whom immediate surgical intervention is not required |
| dasatinib | 7/19/2011 | 11/26/2015 | treatment of adults with newly diagnosed Ph+ CML in chronic phase |
| ofatumumab | 3/9/2012 | n/a | chronic lymphocytic leukemia refractory to fludarabine and alemtuzumab |
| crizotinib | 4/25/2012 | 11/18/2015 | monotherapy in patients with ALK positive locally advanced or metastatic NSCLC |
| remestemcel-L | 5/17/2012 |  | acute graft versus host disease in pediatric patients refractory to treatment with systemic corticosteroid therapy and/or other immunosuppressive agents |
| everolimus | 1/25/2013 | 9/23/2016 | the treatment of adult patients with renal angiomyolipoma associated with tuberous sclerosis complex who do not require immediate surgery |
| brentuximab vedotin | 2/1/2013 | 5/12/2020 | (1) patients with Hodgkin lymphoma after failure of autologous stem cell transplant or after failure of at least two multi-agent chemotherapy regimens in patients who are not ASCT candidates;  (2) patients with systematic anaplastic large cell lymphoma after failure of at least one multi-agent chemotherapy regimen |
| eculizumab | 3/1/2013 | 6/30/2015 | treatment of patients with atypical hemolytic uremic syndrome to reduce complement-mediated thrombotic microangiopathy in children less than 13 years of age and/or weighing less than 40 kg [no conditions for adults] |
| romidepsin | 10/16/2013 | n/a | patients with relapsed/refractory peripheral T-cell lymphoma who are not eligible for transplant and have received at least one prior systemic therapy |
| bosutinib | 3/7/2014 | 8/2/2017 | the treatment of chronic, accelerated, or blast phase Ph+ Chronic Myelogenous Leukemia in adult patients with resistance or intolerance to prior tyrosine kinase inhibitor therapy, and for whom subsequent treatment with imatinib, nilotinib, and dasatinib is not clinically appropriate |
| imatinib mesylate | 4/24/2014 | 5/26/2014 | not specified |
| imatinib mesylate | 7/7/2014 | 7/7/2014 | not specified |
| simeprevir | 1/30/2015 | 11/29/2016 | in combination with sofosbuvir (and without the need for peginterferon alfa and ribavirin) for the treatment of genotype 1 chronic hepatitis C in adults with compensated liver disease |
| dabrafenib | 3/6/2015 | 5/13/2016 | in combination with trametinib for the treatment of patients with unresectable or metastatic melanoma with a BRAF V600 mutation |
| idelalisib | 3/27/2015 | 4/21/2020 | monotherapy for the treatment of patients with follicular lymphoma who have received at least two prior systemic regimens and are refractory to both rituximab and an alkylating agent |
| ceritinib | 3/27/2015 | n/a | anaplastic lymphoma kinase positive locally advanced or metastatic non-small cell lung cancer who have progressed on or who were intolerant to crizotinib |
| ponatinib hydrochloride | 4/2/2015 | n/a | adult patients with chronic phase, accelerated phase, or blast phase chronic myeloid leukemia or Ph+ acute lymphoblastic leukemia for whom other tyrosine kinase inhibitor therapy is not appropriate including if T3151 mutation position or prior TKI resistance or intolerance |
| pembrolizumab | 5/19/2015 | 12/22/2017 | unresectable or metastatic melanoma and disease progression following ipilimumab therapy, and if BRAF V600 mutation positive, following a BRAF or MEK inhibitor |
| ibrutinib | 7/28/2015 | 9/12/2017 | relapsed or refractory mantle cell lymphoma |
| daclatasvir | 8/13/2015 | 11/9/2016 | for use in combination with other agents for the treatment of chronic hepatitis in adult patients with hepatitis C virus genotype 3 and compensated liver disease including cirrhosis |
| asfotase alfa | 8/14/2015 | n/a | enzyme replacement therapy for patients with confirmed diagnosis of paediatric-onset hypophosphatasia |
| blinatumomab | 12/22/2015 | 11/16/2017 | treatment of adults with Philadelphia chromosome-negative relapsed or refractory B precursor acute lymphoblastic leukemia (ALL). |
| deferasirox | 2/24/2016 | 1/10/2017 | (1) the management of chronic iron overload in patients with transfusion dependent anemias aged six years or older (2) the management of chronic iron overload in patients with transfusion dependent anemias aged two to five who cannot be adequately treated with deferoxamine |
| trametinib | 3/11/2016 | 5/13/2016 | in combination with dabrafenib for the treatment of patients with unresectable or metastatic melanoma with a BRAF V600 mutation |
| palbociclib | 3/16/2016 | 11/17/2017 | For use in combination with letrozole for the treatment of postmenopausal women with estrogen-receptor positive human epidermal growth factor receptor 2 negative advanced breast cancer as initial endocrine-based therapy for their metastatic disease |
| pembrolizumab | 4/15/2016 | 9/10/2019 | treatment of patients with metastatic NSCLC whose tumours express PD-L-1 and who have disease progression on or after platinum containing chemotherapy. Patients with EGFR or ALK genomic tumour aberrations should have disease progression on authorized therapy for these aberrations prior to receiving Keytruda. |
| olaparib | 4/29/2016 | 5/2/2018 | Maintenance treatment of adult patients with platinum sensitive relapsed BRCA mutated high grade serous epithelial ovarian, fallopian tube or primary peritoneal cancer who are in response to platinum based chemotherapy |
| nivolumab | 4/29/2016 | 2/21/2018 | treatment of patients with unresectable or metastatic melanoma and disease progression following ipilimumab and, if BRAF V600 positive, a BRAF Inhibitor |
| idarucizumab | 4/29/2016 | 6/28/2018 | antidote specific for dabigatran in patients treated with Pradaza when rapid specific reversal of the anticoagulant effects of dabigatran is required either for emergency surgery/urgent procedures or in situations of life--threatening or uncontrolled bleeding |
| daratumumab | 6/29/2016 | 10/10/2018 | patients with multiple myeloma who have received at least three prior lines of therapy including a proteasome inhibitor and an modulatory agent, or who are refractory to both a PI and an IMiD |
| osimertinib | 7/5/2016 | 1/19/2018 | patients with locally advanced or metastatic EGFR T790M mutation-positive NSCLC who have progressed on or after EGFR TKI therapy. Validated test is required to identify EGFR T790M mutation-positive status prior to treatment |
| alectinib | 9/29/2016 | 9/26/2018 | monotherapy for the treatment of patients with ALK-positive locally advanced or metastatic NSCLC who have progressed or are intolerant to crizotinib |
| venetoclax | 9/30/2016 | 1/13/2020 | monotherapy for the treatment of patients with Chronic lymphocytic leukemia (CLL) with 17p deletion who have received at least one prior therapy or patients with CLL without 17p deletion who have received at least one prior therapy and for whom there are no other available treatment options |
| nivolumab | 10/26/2016 | n/a | (1) treatment of patients with unresectable or metastatic BRAF V600 mutation position melanoma in previously untreated adults;  (2) treatment of patients with unresectable or metastatic melanoma in previously untreated adults when used in combination with ipilimumab |
| agalsidase alfa | 2/10/2017 | n/a | long-term enzyme replacement therapy in patients with confirmed diagnosis of Fabry disease |
| thiotepa | 3/29/2017 | n/a | As part of a high dose chemotherapy consolidation regimen followed by ASTC in adult patients with CNS lymphoma |
| atezolizumab | 4/12/2017 | n/a | patients with locally advanced or metastatic urothelial carcinoma who: Have disease progression during or following platinum-containing chemotherapy; Have disease progression within 12 months of neoadjuvant or adjuvant treatment with platinum containing chemotherapy |
| blinatumomab | 4/28/2017 | n/a | Indicated for the treatment of pediatric patients with Ph- relapsed or refractory B cell precursor ALL |
| obeticholic acid | 5/24/2017 | n/a | primary biliary cholangitis in combination with ursodeoxycholic acid in adults with an inadequate response to UDCA or as monotherapy in adults unable to tolerate UDCA |
| pembrolizumab | 9/8/2017 | n/a | refractory or relapsed classical Hodgkin lymphoma as monotherapy in adults who have failed autologous stem cell transplant and brentouximab vedotin or who are not ASCT candidates and have failed brentouximab vedotin |
| durvalumab for injection | 11/3/2017 | n/a | patients with locally advanced or metastatic urothelial carcinoma who have disease progression during or following platinum containing chemotherapy or have disease progression within 12 months of neoadjuvant or adjuvant treatment with platinum-containing chemotherapy |
| nivolumab | 11/10/2017 | n/a | adult patients with classical Hodgkin lymphoma that has relapsed or progressed after autologous stem cell transplantation and brentuximab vedotin, or 3 or more lines of systemic therapy including ASCT |
| olaratumab | 11/23/2017 | n/a | In combination with doxorubicin for the treatment of adult patients with advanced soft tissue sarcoma not amenable to curative treatment with radiotherapy or surgery and for whom treatment with an anthracycline-containing regimen is appropriate |
| sebelipase alfa | 12/15/2017 | n/a | treatment of lysosomal acid lipase deficiency |
| avelumab | 12/17/2017 | n/a | treatment of patients with metastatic Merkel cell carcinoma in previously treated adults |
| ocrelizumab | 2/14/2018 | n/a | management of adult patients with early primary progressive multiple sclerosis as defined by disease duration and level of disability, in conjunction with imaging features characteristic of inflammatory activity |
| nivolumab | 3/23/2018 | n/a | monotherapy for the treatment of adult patients with advanced (not amenable to curative therapy or local therapeutic measures) or metastatic hepatocellular carcinoma who are intolerant to or have progressed on sorafenib therapy |
| avelumab | 5/3/2018 | 5/14/2019 | locally advanced or metastatic urothelial carcinoma who have received prior platinum-based chemotherapy |
| durvalumad | 5/4/2018 | 8/23/2019 | locally advanced unresectable NSCLC whose disease has not progressed following platinum based chemoradiation therapy |
| olaparib | 5/4/2018 | n/a | monotherapy for the maintenance treatment of adult patients with platinum-sensitive relapsed (PSR) BRCA wild type high-grade epithelial ovarian, fallopian tube, or primary peritoneal cancer who are in response (complete response or partial response) to platinum-based chemotherapy |
| brigatinib | 7/26/2018 | n/a | adult patients with anaplastic lymphoma kinase positive metastatic non-small cell lung cancer who have progressed on or were intolerant to an ALK inhibitor (crizotinib) |
| pembrolizumab | 9/21/2018 | n/a | adult and pediatric patients with relapsed or refractory Primary Mediastinal B-cell Lymphomaor who have relapsed after 2 or more lines of therapy, as monotherapy |
| pralatrexate | 10/26/2018 | n/a | patients with relapsed or refractory peripheral T-cell lymphoma (PTCL) |
| enasidenib (as enasidenib mesylate) | 2/6/2019 | n/a | treatment of adult patients with relapsed or refractory AML with an isocitrate dehydrogenase-2 IHG2 mutation |
| lorlatinib | 2/22/2019 | n/a | monotherapy for the treatment of adult patients with ALK-positive metastatic NSCLC who have progressed on crizotinib and at least one other ALK inhibitor, or patients who have progressed on ceritinib or alectinib |
| cemiplimab | 4/10/2019 | n/a | treatment of adult patients with metastatic or locally advanced cutaneous squamous cell carcinoma who are not candidates for curative surgery or curative radiation |
| pembrolizumab | 4/11/2019 | n/a | treatment of patients with locally advanced unresectable or metastatic urothelial carcinoma, as monotherapy, in adults who are not eligible for cisplatin-containing chemotherapy and whose tumors express PD-L1 as determined by a validated test, or in adults who are not eligible for any platinum-containing chemotherapy regardless of PD-L1 status, |
| pembrolizumab | 4/18/2019 | n/a | adults with (1) unresectable or metastatic MSI-H or dMMR colorectal cancer that has progressed following treatment with a fluoropyrimidine, oxaliplatin, and irinotecan, or (2) MSI-H or dMMR endometrial cancer that has progressed following prior therapy and who have no satisfactory alternative treatment options |
| larotrectinib | 7/10/2019 | n/a | treatment of adult and pediatric patients with solid tumours that have NTRK gene fusion without a known acquired resistance mutation; are metastatic or where surgical resection is likely to result in severe morbidity; and have no satisfactory treatment options |
| atezolizumab | 8/21/2019 | n/a | in combination with nab-paclitaxel, for the treatment of adult patients with unresectable, locally advanced or metastatic triple-negative breast cancer (TNBC) whose tumours have PD-L1 expression ≥ 1%, and who have not received prior chemotherapy for metastatic disease |
| pembrolizumab | 9/20/2019 | n/a | treatment of adult patients, in combination with lenvatinib, with advanced endometrial carcinoma that is not MSI-H or dMMR who have disease progression following prior platinum-based therapy and are not candidates for curative surgery or radiation |
| lenvatinib mesylate | 9/20/2019 | n/a | in combination with pembrolizumab, for the treatment of adult patients with advanced endometrial carcinoma that is not MSI-H or dMMR, who have disease progression following prior platinum-based systemic therapy, and are not candidates for curative surgery or radiation |
| erdafitinib | 10/25/2019 | n/a | adult patients with locally advanced or metastatic urothelial carcinoma whose tumors have susceptible FGFR2 or FGFR3 genetic alterations and; who have disease progression during or following at least one line of prior chemotherapy, including within 12 months of neoadjuvant or adjuvant chemotherapy, |
| avelumab | 11/5/2019 | n/a | treatment of adult patients with metastatic merkel cell carcinoma |
| blinatumomab | 12/19/2019 | n/a | Indicated for the treatment of patients with Philadelphia chromosome-negative CD19 positive B-precursor ALL, in first or second hematologic complete remission with minimal residue disease greater or equal to 0.1%/ treatment of patients with minimal residual disease positive B-cell precursor ALL |
| entrectinib | 2/10/2020 | n/a | the treatment of adult patients with unresectable locally advanced or metastatic extracranial solid tumours, including brain metastases, that have a neurotrophic tyrosine receptor kinase (NTRK) gene fusion without a known acquired resistance mutation, and with no satisfactory treatment options |
| polatuzumab bedotin | 7/9/2020 | n/a | in combination with bendamustine and rituximab for the treatment of adult patients with relapsed or refractory diffuse large B-cell lymphoma, not otherwise specified, who are not eligible for ASCT and have received at least one prior therapy |
| remdesivir | 7/27/2020 | n/a | for the treatment of coronavirus disease 2019 (COVID-19) in adults and adolescents (aged 12 years and older with body weight at least 40 kg) with pneumonia requiring supplemental oxygen |
| pembrolizumab | 12/14/2020 | n/a | For the treatment of adult patients with bacillus Calmette-guerin-unresponsive, high risk, non-muscle invasive bladder cancer with carcinoma in-situ with or without papillary tumours eligible for or have elected not to undergo cystectomy |
| Bacillus Calmette-Guérin (BCG): Russian BCG-I (Russian or Moscow) strain | 12/24/2020 | n/a | adjuvant therapy after transurethral resection of a primary or relapsing superficial papillary urothelial cell carcinoma of the bladder stage Ta (grade 2 or 3) or T1 (grade 1, 2, or 3), without concomitant carcinoma in situ. It is only recommended for stage Ta grade 1 papillary tumors, when there is judged to be a high risk (>50%) of tumor recurrence. |
| pembrolizumab | 2/5/2021 | n/a | the treatment of adult and pediatric patients with refractory or relapsed classical Hodgkin Lymphoma who have failed ASCT, or who are not candidates for multi-agent salvage chemotherapy and ASCT, |
| nivolumab | 2/11/2021 | n/a | In combination with ipilimumab for the treatment of adult patients with MSI-H or dMMR metastatic colorectal cancer after prior fuoropyrimidine-based therapy in combination with oxaplatin or irinotecan |
| trastuzumab Deruxtecan | 4/15/2021 | n/a | treatment of adult patients with unresectable or metastatic HER2-positive breast cancer who have received prior treatment with trastuzumab emtansine |
| idecabtagene vicleucel | 5/26/2021 | n/a | adult patients with multiple myeloma who have received at least three prior therapies, including an immunomodulatory agent, a proteasome inhibitor, and an anti-CD38 antibody and who are refractory to their last treatment |
| tepotinib | 5/27/2021 | n/a | adult patients with locally advanced or metastatic non-small cell lung cancer harbouring mesenchymal-epithelial transition tyrosine kinase receptor exon 14 skipping alterations |
| selpercatinib | 6/15/2021 | n/a | monotherapy for the treatment of metastatic RET fusion-positive NSCLC in adult patients, RET-mutant medullary thyroid cancer in adult and pediatric patients 12 years of age and older with unresectable advanced or metastatic disease, RET fusion-positive differentiated thyroid carcinoma in adult patients with advanced or metastatic disease (not amenable to surgery or radioactive iodine therapy) following prior treatment with sorafenib and/or lenvatinib |
| pralsetinib | 6/30/2021 | n/a | the treatment of adult patients with RET fusion-positive locally advanced unresectable or metastatic NSCLC |

ALK: anaplastic lymphoma kinase; ALL: acute lymphoblastic leukemia; ARV: Antiretroviral; BRAF: ASCT: autologous stem cell transplant; B-Raf proto-oncogene; BRCA: breast cancer gene; CML: Chronic myelogenous leukemia; CNS: central nervous system; dMMRL deficient mismatch repair; ECOG: Eastern Cooperative Oncology Group; EGFR: epidermal growth factor receptor; GBM: glioblastoma; GIST: gastrointestinal stromal tumour; HBV: hepatitis B; HER2: human epidermal growth factor receptor 2; iMiD: immunomodulatory drugs; KRAS: Kirsten rat sarcoma virus; MEK: mitogen-activated protein kinase; MSI-H: Microsatellite Instability High; NTRK: neurotrophic tyrosine receptor kinase; NSCLC: non small cell lung cancer; PA: psudeomonas aeruginosa; PD-L1: programmed death-ligand 1; Ph+: Philadelphia chromosome positive; Ph-: Philadelphia chromosome negative; PI: proteasome inhibitors; T-ALL: T-cell acute lymphoblastic leukemia; TKI: tyrosine kinase inhibitors; UDCA: ursodeoxycholic acid

# Supplementary Table 2: Total Oncology Indications Approved under NOC/c (January 1,1998-June 30, 2021)

| **Active ingredient** | **NOC/c granted** | **Indication** | **Regulatory Status in Canada (8/30/2021)*** |
| --- | --- | --- | --- |
| 1. imatinib | 9/20/2001 | Adult patients with Ph+ CML in blast crisis, accelerated phase or in chronic phase (after failure of interferon-ax therapy). | transferred |
| 1. bicalutamide | 2002-11-25 | for immediate therapy in some patients with localized (T1-T2) prostate cancer who are inappropriate for surgery or radiotherapy | Suspended 8/13/2003 |
| 1. imatinib | 10/8/2003 | Adult patients with newly diagnosed Ph+ CML | transferred |
| 1. gefitinib | 12/17/2003 | third line treatment of patients with locally advanced or metastatic NSCLC after failure of two prior chemotherapy regimens | transferred |
| 1. anastrozole | 6/30/2004 | adjuvant treatment of postmenopausal women with hormone receptor positive early breast cancer | transferred |
| 1. bortezomib | 1/27/2005 | treatment of multiple myeloma patients who have relapsed following front-line therapy and refractory to their most recent therapy. | transferred |
| 1. letrozole | 4/1/2005 | for the extended adjuvant treatment of early breast cancer in post-menopausal women who have received prior standard adjuvant tamoxifen therapy | transferred |
| 1. capecitabine | 12/7/2005 | adjuvant treatment of patients with Stage III (Dukes' stage C) colon cancer | transferred |
| 1. exemestane | 5/12/2006 | adjuvant treatment of early breast cancer | transferred |
| 1. sorafenib tablets | 7/28/2006 | treatment of locally advanced/metastatic renal cell carcinoma in patients who failed prior cytokine therapy or are considered unsuitable for such therapy | transferred |
| 1. sunitinab malate | 8/17/2006 | treatment of metastatic renal cell carcinoma of clear cell histology after failure of cytokine-based therapy or in patients who are considered likely to be intolerant of such therapy | transferred |
| 1. letrozole | 10/6/2006 | for the adjuvant treatment of post-menopausal women with hormone receptor positive early breast cancer | transferred |
| 1. docetaxel | 12/14/2006 | adjuvant treatment of patients with operable node-positive breast cancer, in combination with doxorubicin and cyclophosphamide | transferred |
| 1. dasatinib | 3/26/2007 | treatment of adults with chronic, accelerated or blast phase CML with resistance or intolerance to prior therapy including imatinib mesylate | transferred |
| 1. imatinib mesylate | 5/24/2007 | Treatment of pediatric patients with newly diagnosed Ph+ CML in chronic phase | transferred |
| 1. nelarabine | 9/22/2007 | Patients with refractory or recurrent T-ALL or T-lymphoblastic lymphoma | transferred |
| 1. panitumumab | 4/3/2008 | monotherapy for the treatment of patients with EGFR expressing metastatic colorectal carcinoma with non-mutated (wild type) KRAS after failure of fluoropyrimidine-, oxaliplatin- and irinotecan-containing chemotherapy regimens | transferred |
| 1. sunitinab malate | 5/1/2008 | treatment of metastatic renal cell carcinoma of clear cell histology | transferred |
| 1. nilotinib | 9/9/2008 | accelerated phase Ph+ CML in adult patients resistant to or intolerant of at least one prior therapy including imatinib | transferred |
| 1. bevacizumab | 2/6/2009 | used in combination with paclitaxel for treatment of patients with HER2 negative breast cancer and who are ECOG class 0-1 | Suspended 11/25/2011 |
| 1. imatinib mesylate | 12/22/2009 | For the adjuvant treatment of adult patients who are at intermediate to high risk of relapse following complete resection of Kit (CD117) positive GIST | transferred |
| 1. bevacizumab | 3/24/2010 | for the treatment of patients with GBM after relapse or disease progression | Withdrawn 5/23/2018 |
| 1. nilotinib | 7/22/2010 | treatment of chronic phase Ph+ CML in adult patients resistant to or intolerant of at least one prior therapy including imatinib | transferred |
| 1. nilotinib | 6/23/2011 | treatment of adult patients with newly diagnosed Ph+ CML in chronic phase | transferred |
| 1. everolimus | 6/30/2011 | for the treatment of patients of 3 years of age or older with subependymal giant cell astrocytoma (SEGA) associated with tuberous sclerosis complex that have demonstrated serial growth who are not candidates for surgical resection and for whom immediate surgical intervention is not required | transferred |
| 1. dasatinib | 7/19/2011 | treatment of adults with newly diagnosed Ph+ CML in chronic phase | transferred |
| 1. ofatumumab | 2012-03-09 | chronic lymphocytic leukemia refractory to fludarabine and alemtuzumab | Discontinued 3/11/2019 |
| 1. everolimus | 1/25/2013 | the treatment of adult patients with renal angiomyolipoma associated with tuberous sclerosis complex who do not require immediate surgery | transferred |
| 1. brentuximab vedotin | 2/1/2013 | (1) patients with Hodgkin lymphoma after failure of ASCT or after failure of at least two multi-agent chemotherapy regimens in patients who are not ASCT candidates;  (2) patients with systematic anaplastic large cell lymphoma after failure of at least one multi-agent chemotherapy regimen | transferred |
| 1. romidepsin | 10/16/2013 | patients with relapsed/refractory peripheral T-cell lymphoma who are not eligible for transplant and have received at least one prior systemic therapy | active |
| 1. bosutinib | 3/7/2014 | the treatment of chronic, accelerated, or blast phase Ph+ CML in adult patients with resistance or intolerance to prior tyrosine kinase inhibitor therapy, and for whom subsequent treatment with imatinib, nilotinib, and dasatinib is not clinically appropriate | transferred |
| 1. dabrafenib | 3/6/2015 | in combination with trametinib for the treatment of patients with unresectable or metastatic melanoma with a BRAF V600 mutation | transferred |
| 1. idelalisib | 3/27/2015 | monotherapy for the treatment of patients with follicular lymphoma who have received at least two prior systemic regimens and are refractory to both rituximab and an alkylating agent | transferred |
| 1. ceritinib | 3/27/2015 | ALK+ locally advanced or metastatic NSCLC who have progressed on or who were intolerant to crizotinib | active |
| 1. ponatinib hydrochloride | 4/2/2015 | adult patients with chronic phase, accelerated phase, or blast phase CML or Ph+ ALL for whom other tyrosine kinase inhibitor therapy is not appropriate including if T3151 mutation position or prior TKI resistance or intolerance | active |
| 1. pembrolizumab | 5/19/2015 | unresectable or metastatic melanoma and disease progression following ipilimumab therapy, and if BRAF V600 mutation positive, following a BRAF or MEK inhibitor | transferred |
| 1. ibrutinib | 7/28/2015 | relapsed or refractory mantle cell lymphoma | transferred |
| 1. blinatumomab | 12/22/2015 | treatment of adults with Philadelphia chromosome-negative relapsed or refractory B precursor acute lymphoblastic leukemia. | transferred |
| 1. trametinib | 3/11/2016 | in combination with dabrafenib for the treatment of patients with unresectable or metastatic melanoma with a BRAF V600 mutation | transferred |
| 1. palbociclib | 3/16/2016 | For use in combination with letrozole for the treatment of postmenopausal women with estrogen-receptor positive human epidermal growth factor receptor 2 negative advanced breast cancer as initial endocrine-based therapy for their metastatic disease | transferred |
| 1. pembrolizumab | 4/15/2016 | treatment of patients with metastatic NSCLC whose tumours express PD-L1 and who have disease progression on or after platinum containing chemotherapy. Patients with EGFR or ALK genomic tumour aberrations should have disease progression on authorized therapy for these aberrations prior to receiving Keytruda. | transferred |
| 1. olaparib | 4/29/2016 | Maintenance treatment of adult patients with platinum sensitive relapsed BRCA mutated high grade serous epithelial ovarian, fallopian tube or primary peritoneal cancer who are in response to platinum based chemotherapy | transferred |
| 1. nivolumab | 4/29/2016 | treatment of patients with unresectable or metastatic melanoma and disease progression following ipilimumab and, if BRAF V600 positive, a BRAF Inhibitor | transferred |
| 1. daratumumab | 6/29/2016 | patients with multiple myeloma who have received at least three prior lines of therapy including a proteasome inhibitor and an modulatory agent, or who are refractory to both a PI and an IMiD | transferred |
| 1. osimertinib | 7/5/2016 | patients with locally advanced or metastatic EGFR T790M mutation-positive NSCLC who have progressed on or after EGFR TKI therapy. Validated test is required to identify EGFR T790M mutation-positive status prior to treatment | transferred |
| 1. alectinib | 9/29/2016 | monotherapy for the treatment of patients with ALK-positive locally advanced or metastatic NSCLC who have progressed or are intolerant to crizotinib | transferred |
| 1. venetoclax | 9/30/2016 | monotherapy for the treatment of patients with CLL with 17p deletion who have received at least one prior therapy or patients with CLL without 17p deletion who have received at least one prior therapy and for whom there are no other available treatment options | transferred |
| 1. nivolumab | 10/26/2016 | (1) treatment of patients with unresectable or metastatic BRAF V600 mutation position melanoma in previously untreated adults;  (2) treatment of patients with unresectable or metastatic melanoma in previously untreated adults when used in combination with ipilimumab | transferred |
| 1. thiotepa | 3/29/2017 | As part of a high dose chemotherapy consolidation regimen followed by ASCT in adult patients with CNS lymphoma | active |
| 1. atezolizumab | 4/12/2017 | patients with locally advanced or metastatic urothelial carcinoma who: Have disease progression during or following platinum-containing chemotherapy; Have disease progression within 12 months of neoadjuvant or adjuvant treatment with platinum containing chemotherapy, | active |
| 1. blinatumomab | 4/28/2017 | Indicated for the treatment of pediatric patients with Ph- relapsed or refractory B cell precursor ALL | active |
| 1. pembrolizumab | 9/8/2017 | refractory or relapsed classical Hodgkin lymphoma as monotherapy in adults who have failed ASCT and brentuximab vedotin or who are not ASCT candidates and have have failed brentuximab vedotin | withdrawn 02/03/2021 |
| 1. durvalumab | 11/3/2017 | patients with locally advanced or metastatic urothelial carcinoma who have disease progression during or following platinum containing chemotherapy or have disease progression within 12 months of neoadjuvant or adjuvant treatment with platinum-containing chemotherapy | active |
| 1. nivolumab | 11/10/2017 | adult patients with classical Hodgkin Lymphoma that has relapsed or progressed after autologous stem cell transplantation and brentuximab vedotin, or 3 or more lines of systemic therapy including ASCT | active |
| 1. olaratumab | 11/23/2017 | In combination with doxorubicin for the treatment of adult patients with advanced soft tissue sarcoma not amenable to curative treatment with radiotherapy or surgery and for whom treatment with an anthracycline-containing regimen is appropriate | discontinued 10/8/2020 |
| 1. avelumab | 12/17/2017 | treatment of patients with metastatic Merkel cell carcinoma in previously treated adults | transferred |
| 1. nivolumab | 3/23/2018 | monotherapy for the treatment of adult patients with advanced (not amenable to curative therapy or local therapeutic measures) or metastatic hepatocellular carcinoma who are intolerant to or have progressed on sorafenib therapy | active |
| 1. avelumab | 5/3/2018 | locally advanced or metastatic urothelial carcinoma who have received prior platinum-based chemotherapy | transferred |
| 1. durvalumad | 5/4/2018 | locally advanced unresectable NSCLC whose disease has not progressed following platinum based chemoradiation therapy | transferred |
| 1. olaparib | 5/4/2018 | monotherapy for the maintenance treatment of adult patients with platinum-sensitive relapsed BRCA wild type high-grade epithelial ovarian, fallopian tube, or primary peritoneal cancer who are in response (complete response or partial response) to platinum-based chemotherapy | active |
| 1. brigatinib | 7/26/2018 | adult patients with ALK positive metastatic NSCLC who have progressed on or were intolerant to an ALK inhibitor (crizotinib) | active |
| 1. pembrolizumab | 9/21/2018 | adult and pediatric patients with relapsed or refractory Primary Mediastinal B-cell Lymphoma or who have relapsed after 2 or more lines of therapy, as monotherapy | active |
| 1. pralatrexate | 10/26/2018 | patients with relapsed or refractory peripheral T-cell lymphoma | active |
| 1. enasidenib (as enasidenib mesylate) | 2/6/2019 | treatment of adult patients with relapsed or refractory AML with an isocitrate dehydrogenase-2 IHG2 mutation | active |
| 1. lorlatinib | 2/22/2019 | monotherapy for the treatment of adult patients with ALK-positive metastatic NSCLC who have progressed on crizotinib and at least one other ALK inhibitor, or patients who have progressed on ceritinib or alectinib | active |
| 1. cemiplimab | 4/10/2019 | treatment of adult patients with metastatic or locally advanced cutaneous squamous cell carcinoma who are not candidates for curative surgery or curative radiation | active |
| 1. pembrolizumab | 4/11/2019 | treatment of patients with locally advanced unresectable or metastatic urothelial carcinoma, as monotherapy, in adults who are not eligible for cisplatin-containing chemotherapy and whose tumors express PD-L1 as determined by a validated test, or in adults who are not eligible for any platinum-containing chemotherapy regardless of PD-L1 status | active |
| 1. pembrolizumab | 4/18/2019 | adults with (1) unresectable or metastatic MSI-H or dMMR colorectal cancer that has progressed following treatment with a fluoropyrimidine, oxaliplatin, and irinotecan, or (2) MSI-H or dMMR endometrial cancer that has progressed following prior therapy and who have no satisfactory alternative treatment options | active |
| 1. larotrectinib | 7/10/2019 | treatment of adult and pediatric patients with solid tumours that have NTRK gene fusion without a known acquired resistance mutation; are metastatic or where surgical resection is likely to result in severe morbidity; and have no satisfactory treatment options | active |
| 1. atezolizumab | 8/21/2019 | in combination with nab-paclitaxel, for the treatment of adult patients with unresectable, locally advanced or metastatic triple-negative breast cancer (TNBC) whose tumours have PD-L1 expression ≥ 1%, and who have not received prior chemotherapy for metastatic disease | active |
| 1. pembrolizumab | 9/20/2019 | treatment of adult patients, in combination with lenvatinib, with advanced endometrial carcinoma that is not MSI-H or dMMR who have disease progression following prior platinum-based therapy and are not candidates for curative surgery or radiation | active |
| 1. lenvatinib mesylate | 9/20/2019 | in combination with pembrolizumab, for the treatment of adult patients with advanced endometrial carcinoma that is not MSI-H or dMMR, who have disease progression following prior platinum-based systemic therapy, and are not candidates for curative surgery or radiation | active |
| 1. erdafitinib | 10/25/2019 | adult patients with locally advanced or metastatic urothelial carcinoma whose tumors have susceptible FGFR2 or FGFR3 genetic alterations and; who have disease progression during or following at least one line of prior chemotherapy, including within 12 months of neoadjuvant or adjuvant chemotherapy, | active |
| 1. avelumab | 11/5/2019 | treatment of adult patients with metastatic merkel cell carcinoma | transferred |
| 1. blinatumomab | 12/19/2019 | the treatment of patients with Ph- CD19 positive B-precursor ALL, in first or second hetologic complete remission with minimal residue disease greater or equal to 0.1%/ treatment of patients with minimal residual disease positive B-cell precursor ALL | active |
| 1. entrectinib | 2/10/2020 | the treatment of adult patients with unresectable locally advanced or metastatic extracranial solid tumours, including brain metastases, that have a NTRK gene fusion without a known acquired resistance mutation, and with no satisfactory treatment options | active |
| 1. polatuzumab bedotin | 7/9/2020 | in combination with bendamustine and rituximab for the treatment of adult patients with relapsed or refractory diffuse large B-cell lymphoma, not otherwise specified, who are not eligible for ASCT and have received at least one prior therapy | active |
| 1. pembrolizumab | 12/14/2020 | Treatment of adult patients with bacillus Calmette Guerin unresponsive, high risk, non-muscle invasive bladder cancer with carcinoma in situ with or without papilary tumours who are eligible for or have elected not to undergo cystectomy | active |
| 1. Bacillus Calmette-Guérin (BCG): Strain Russian BCG-I | 12/24/2020 | adjuvant therapy after transurethral resection of a primary or relapsing superficial papillary urothelial cell carcinoma of the bladder stage Ta (grade 2 or 3) or T1 (grade 1, 2, or 3), without concomitant carcinoma in situ. It is only recommended for stage Ta grade 1 papillary tumors, when there is judged to be a high risk (>50%) of tumor recurrence. | active |
| 1. pembrolizumab | 2/5/2021 | the treatment of adult and pediatric patients with refractory or relapsed classical Hodgkin Lymphoma who have failed ASCT, or who are not candidates for multi-agent salvage chemotherapy and ASCT. | active |
| 1. nivolumab | 2/11/2021 | n combination with ipilimumab, for the treatment of adult patients with MSI-H or dMMR metastatic colorectal cancer after prior fluoropyrimidine-based therapy in combination with oxaliplatin or irinotecan | active |
| 1. trastuzumab deruxtecan | 4/15/2021 | treatment of adult patients with unresectable or metastatic HER2-positive breast cancer who have received prior treatment with trastuzumab emtansine. | active |
| 1. idecabtagene vicleucel | 5/26/2021 | adult patients with multiple myeloma who have received at least three prior therapies, including an immunomodulatory agent, a proteasome inhibitor, and an anti-CD38 antibody and who are refractory to their last treatment. | active |
| 1. tepotinib | 5/27/2021 | adult patients with locally advanced or metastatic non-small cell lung cancer harbouring mesenchymal-epithelial transition tyrosine kinase receptor exon 14 skipping alterations | active |
| 1. selpercatinib | 6/15/2021 | monotherapy for the treatment of metastatic RET fusion-positive NSCLC in adult patients, RET-mutant medullary thyroid cancer in adult and pediatric patients 12 years of age and older with unresectable advanced or metastatic disease, RET fusion-positive differentiated thyroid carcinoma in adult patients with advanced or metastatic disease (not amenable to surgery or radioactive iodine therapy) following prior treatment with sorafenib and/or lenvatinib | active |
| 1. pralsetinib | 6/30/2021 | the treatment of adult patients with rearranged during transfection fusion-positive locally advanced unresectable or metastatic NSCLC | active |

AA: Accelerated Approval; ALK: anaplastic lymphoma kinase; ALL: acute lymphoblastic leukemia; ARV: Antiretroviral; BRAF: ASCT: autologous stem cell transplant; B-Raf proto-oncogene; BRCA: breast cancer gene; CML: Chronic myelogenous leukemia; CNS: central nervous system; dMMRL deficient mismatch repair; ECOG: Eastern Cooperative Oncology Group; EGFR: epidermal growth factor receptor; GBM: glioblastoma; GIST: gastrointestinal stromal tumour; HBV: hepatitis B; HER2: human epidermal growth factor receptor 2; iMiD: immunomodulatory drugs; KRAS: Kirsten rat sarcoma virus; MEK: mitogen-activated protein kinase; MSI-H: Microsatellite Instability High; NTRK: neurotrophic tyrosine receptor kinase; NSCLC: non small cell lung cancer; PA: psudeomonas aeruginosa; PD-L1: programmed death-ligand 1; Ph+: Philadelphia chromosome positive; Ph-: Philadelphia chromosome negative; PI: proteasome inhibitors; T-ALL: T-cell acute lymphoblastic leukemia; TKI: tyrosine kinase inhibitors; UDCA: ursodeoxycholic acid

* Active: conditions have not yet been met; Discontinued: the manufacturer stops selling the drug for business reasons; Transferred: conditions have been met and the NOC/c has been transferred to a NOC; Suspended: Health Canada suspended the NOC for safety reasons; Withdrawn: a specific indication is withdrawn by the manufacturer without affecting other approved indications.

# Table 3: Eligibility criteria for oncology indications approved under NOC/c policy

| **Drug/Indication** | **Eligibility rationale & source (SBD/RDS)** | **Categorization** |
| --- | --- | --- |
| 1. Palbociclib (in combination with letrozole for ER+/ HER2- breast cancer) | - “benefits of Ibrance therapy in combination with letrozole are considered to potentially provide a significant increase in efficacy such that the overall benefit/risk profile is improved over the existing therapy of letrozole alone” (SBD) - “Ibrance, in combination with letrozole, has the potential to provide a significant increase in efficacy… over the existing therapy letrozole” (RDS) | Blended |
| 1. Polatuzumab vedotin (DLBCL) | - “the drug has the potential to provide an effective treatment of a disease that is not adequately managed by other drugs” (SBD) - “the standard treatment option for patients who have relapsed or refractory DLBCL and are not eligible for an autologous stem cell transplant include combination chemotherapy regimens…[h]owever, even with these therapies, the prognosis is poor for these patients” (SBD) | Blended |
| 1. Pralatrexate (peripheral T-cell lymphoma) | - “there is currently no widely accepted standard of care” (SBD) - “agents currently available to treat peripheral T-cell lymphoma patients in the relapsed or refractory setting include romidepsin… and brentuximab vedotin” (SBD) | Blended |
| 1. Alectinib (ALK+ NSCLC) | - “condition not adequately managed by available therapies” (SBD) - “potential to provide a significant increase in efficacy and/or decrease in risk such that the overall benefit/risk profile is improved over existing therapies” (RDS) | Improvement over existing therapy |
| 1. Brigatinib (ALK+ NSCLC) | - “substantial improvement over existing therapies” and “offers a considerable improvement in the overall benefit/risk profile over existing treatment options” (SBD) | Improvement over existing therapy |
| 1. Erdafitinib (urothelial carcinoma) | - “promising evidence that the benefit-risk profile of Balversa would be improved over existing therapies”(SBD) | Improvement over existing therapy |
| 1. Lorlatinib (ALK+ NSCLC) | - “potential to provide an improvement in the benefit-risk profile over existing therapies for…a disease that is not adequately managed by a drug marketed in Canada” (SBD) | Improvement over existing therapy |
| 1. Pembrolizumab (melanoma) | - “potential to provide a significant increase in efficacy such that the overall benefit/risk profile is improved over existing therapies for a disease or condition that is not adequately managed by a drug marketed in Canada” (SBD) | Improvement over existing therapy |
| 1. Avelumab (Ph- CD 19+ B-precursor ALL) | - “given the lack of therapeutic options available to patients within the indication, the evidence provided in support of the efficacy of Bavencio is considered promising” (SBD) | No existing therapy – lower standard |
| 1. Blinatumomab (adult Ph- B precursor ALL) | - “adult patients have extremely poor long-term outcomes, and very limited treatment options which include aggressive chemotherapy regimens that are generally cytotoxic and may be poorly tolerated” (SBD) - “there are very few treatment options” (RDS) - “a favourable benefit/risk profile…meets the NOC/c criteria of demonstrating a promising clinical benefit for patients with a serious, life-threatening disease who are unresponsive to or unable to tolerate existing therapies” (RDS) | No existing therapy – lower standard |
| 1. Bosutinib (Ph+ chronic myelogenous leukemia) | - “Bosulif does fulfil an unmet medical need as an alternate treatment option for CML patients who do not respond to or who are intolerant of currently marketed authorized TKIs in Canada” (SBD) | No existing therapy – lower standard |
| 1. Brentuximab vedotin (Hodgkin Lymphoma) | - “few treatment options are available for relapsed or refractory HL in patients who have undergone an ASCT, or are not candidates for an ASCT” (SBD) - “based on the primary endpoint (ORR) results… Adcetris therapy is considered likely to be efficacious in the population in which it was tested” (SBD) | No existing therapy – lower standard |
| 1. Brentuximab vedotin (systemic anaplastic large cell lymphoma) | - “Currently, sALCL patients who have failed frontline therapies represent an unmet medical need” (SBD) - “Adcetris therapy is considered likely to be efficacious in the population in which it was tested” (SBD) | No existing therapy – lower standard |
| 1. Crizotinib (ALK+ NSCLC) | - “The lack of effective systemic therapies for patients with this rare disease…constitutes an unmet medical need which Xalkori appears to fulfill” (SBD) | No existing therapy – lower standard |
| 1. Daratumumab (multiple myeloma) | - “following several relapses, very limited therapeutic options remain for these heavily pre-treated and refractory patients’ therefore, there remains a high unmet medical need for these patients” (SBD) - “once these patients relapsed or become refractory to available therapeutic options, limited treatment options remain” (RDS) | No existing therapy – lower standard |
| 1. Ibrutinib (mantle cell lymphoma) | - “in Canada, approved treatment options are limited and include chlorambucil and bortezomib” (RDS) | No existing therapy – lower standard |
| 1. Nelarabine (T-cell acute lymphoblastic leukemia and lymphoblastic lymphoma) | - “T-cell acute lymphoblastic leukemia and lymphoblastic lymphoma are both aggressive cancers with very few treatment options” (SBD) - “patients who have relapsed or are refractory to two or more prior induction regimens have no standard, proven treatment options” (SBD) - “refractory or relapsed T-cell LBL and ALL in pediatric and adult patients in an aggressive cancer with no real treatment options” (SBD) | No existing therapy – lower standard |
| 1. Nivolumab (hepatocellular carcinoma) | - “given the information provided in the submission and the lack of therapies for patients with this serious life-threatening disease, Opdivo is considered to have a positive benefit/risk profile” (RDS) | No existing therapy – lower standard |
| 1. Nivolumab (classical Hodgkin lymphoma) | - “Promising efficacy of Opdivo in treatment of refractory, relapsed cHL patients, who have unmet medical needs, has been demonstrated” (RDS) | No existing therapy – lower standard |
| 1. Olaratumab (advanced soft tissue sarcoma) | - “new treatments in first and subsequent lines of therapy are required for patients” (SBD) | No existing therapy – lower standard |
| 1. Pembrolizumab (classical hodgkin lymphoma) | - “promising efficacy of Keytruda…in treatment of adult patients with refractory, relapsed cHL, who have unmet medical needs” (RDS) | No existing therapy – lower standard |
| 1. Pembrolizumab (NSCLC with PD-L1 expressing tumours) | - “patients who experienced a disease progression have limited therapeutic options” (RDS) | No existing therapy – lower standard |
| 1. Venetoclax (CLL with 17p deletion) | - “previously-treated CLL [is] a serious disease for which there is an unmet medical need” (SBD) | No existing therapy – lower standard |
| 1. Atezolizumab (urothelial carcinoma) | - “there are no authorized agents available to patients” (SBD) - “given the lack of therapeutic options available to patients within the indication…the tentative benefit-risk balance can be considered favourable” (SBD) - “there are few treatment options…[n]o treatments have been previously authorized in this setting in Canada” (RDS) | No existing therapy – strict standard |
| 1. Avelumab (merkel cell carcinoma) | - “promising evidence of clinical effectiveness in…a population with an unmet medical need” (SBD) - “there are no approved treatment options” (RDS) | No existing therapy – strict standard |
| 1. Blinatumomab (pediatric Ph- B-cell ALL) | - “no effective treatment options” (RDS) | No existing therapy – strict standard |
| 1. Cemipilimab (cutaneous squamous cell carcinoma) | - “for this small subset of patients, no approved treatments are currently available and there is no standard of care” (SBD) - “Libtayo has the potential to provide effective treatment of … a disease for which no drug is presently marketed in Canada” (SBD) | No existing therapy – strict standard |
| 1. Ceritinib (ALK+ NSCLC) | - “patients whose disease has progressed on crizotinib represent a population with an unmet medical need. Zykadia represents a new therapeutic option for these patients” (SBD) - “the drug has the potential to provide effective treatment of a disease or condition for which no drug is presently marketed in Canada” (SBD) | No existing therapy – strict standard |
| 1. Durvalumab (urothelial carcinoma) | - “at the time of advanced consideration under the NOC/c policy was granted, there were no authorized agents available to patients” (SBD) - “the drug has potential to provide effective treatment, prevention or diagnosis of a disease or condition for which no drug is presently marketed in Canada” (SBD) - “the promising evidence of efficacy is considered to outweigh the potential risks of Imfinzi in the context of a disease for which no other treatment is available” (SBD) - “at the time Imfinzi was granted advanced consideration in accordance with the guidance document: Notice of compliance with Conditions (NOC/c), treatment options were limited to cytotoxic chemotherapy salvage regimes, which are not proven to improve patients’ quality of life or extend survival… a large proportion of patients in this setting are unable to receive further treatment with cytotoxic chemotherapies leaving best supportive care of clinical trials as the only available option” | No existing therapy – strict standard |
| 1. Enasidenib (acute myeloid leukemia with an IDH2 mutation) | - “relapsed/refractory acute myeloid leukemia remains an area of high unmet medical need” (SBD) - “at the time of submission, there were no approved therapies for the specific indication” | No existing therapy – strict standard |
| 1. Entrectinib (solid tumours with NTRK gene fusion) | - “there are few, if any, treatment options available for this patient population if standard treatments have failed” (SBD) - “the drug is intended to treat a patient population with a life-threatening disease, for which no drug was marketed at the time of the request” | No existing therapy – strict standard |
| 1. Osimertinib (EGFR T790M mutation positive NSCLC) | - “the safety risks associated with Tagrisso are outweighed by the clinically meaningful benefit of Tagrisso in this patient population with limited treatment options, thereby fulfilling an unmet medical need” (SBD) - “there are no market authorized therapies that target the acquired EGFR T790M TKI-resistance conferring mutation” (SBD) - “there are no market authorized therapies that have demonstrated efficacy against the acquired T790M TKI-resistance-conferring mutation” (RDS) - “while the risks of toxicity with Tagrisso are substantial, they are outweighed by the clinical benefit of Tagrisso in this patient population with very limited effective treatment options” (RDS) | No existing therapy – strict standard |
| 1. Panitumumab (EGFR expressing metastatic colorectal carcinoma with wild type KRAS) | - “there are no treatments available in Canada…and these patients have limited options available to them for further treatment. Vectibix…has the potential to address this unmet medical need” (SBD) - “patients…only have the option for palliative therapy. Due to the current unmet needs for the treatment… an NOC under the NOC/c Policy was granted” (SBD) | No existing therapy – strict standard |

ALL: acute lymphoblastic leukaemia; ALK+: anaplastic lymphoma kinase positive; ASCT: autologous stem cell transfer; cHL: classical Hodgkins lymphoma; CLL: chronic lymphocytic leukemia; DLBCL: diffuse large b-cell lymphoma; EGFR: epidermal growth factor receptor; ER+: estrogen receptor positive; HER2-: human epidermal growth factor receptor 2 negative; IDH2: Isocitrate dehydrogenase; KRAS: Kirsten rat sarcoma virus; LBL: lymphoblastic leukaemia; NSCLC: non small cell lung cancer; NTRK: neurotrophic tyrosine receptor kinase; ORR: objective response rate; PD-L1: Programmed Cell Death Ligand 1; Ph+: Philadelphia chromosome positive; Ph- : Philadelphia chromosome negative; RDS: Regulatory Decision Summary; SBD: Summary Basis of Decision; T-ALL: T-cell acute lymphoblastic leukaemia; TKI: tyrosine kinase inhibitor.
